# Systemic miRNA-7 delivery inhibits tumor angiogenesis and growth in murine xenograft glioblastoma

**DOI:** 10.18632/oncotarget.2235

**Published:** 2014-07-18

**Authors:** Negar Babae, Meriem Bourajjaj, Yijia Liu, Judy R. Van Beijnum, Francesco Cerisoli, Puthupparampil V. Scaria, Mark Verheul, Maaike P. Van Berkel, Ebel H. E. Pieters, Rick J. Van Haastert, Afrouz Yousefi, Enrico Mastrobattista, Gert Storm, Eugene Berezikov, Edwin Cuppen, Martin Woodle, Roel Q. J. Schaapveld, Gregoire P. Prevost, Arjan W. Griffioen, Paula I. Van Noort, Raymond M. Schiffelers

**Affiliations:** ^1^ Utrecht Institute for Pharmaceutical Sciences, University Utrecht, Utrecht, the Netherlands; ^2^ InteRNA Technologies B.V., Utrecht, the Netherlands; ^3^ Aparna Biosciences Corporation, Rockville MD, USA; ^4^ VU University Medical Center, MB Amsterdam, the Netherlands; ^5^ MIRA Institute for Biomedical Technology & Technical Medicine, Faculty of Science & Technology, University of Twente, AE Enschede, the Netherlands; ^6^ Hubrecht Institute, Cancer Genomics Center and University Medical Center Utrecht, Utrecht, the Netherlands; ^7^ Laboratory Clinical Chemistry & Haematology, University Medical Center Utrecht (UMCU), Utrecht, the Netherlands

**Keywords:** microRNA, miR-7, angiogenesis, delivery, Therapy

## Abstract

Tumor-angiogenesis is the multi-factorial process of sprouting of endothelial cells (EC) into micro-vessels to provide tumor cells with nutrients and oxygen. To explore miRNAs as therapeutic angiogenesis-inhibitors, we performed a functional screen to identify miRNAs that are able to decrease EC viability. We identified miRNA-7 (miR-7) as a potent negative regulator of angiogenesis. Introduction of miR-7 in EC resulted in strongly reduced cell viability, tube formation, sprouting and migration. Application of miR-7 in the chick chorioallantoic membrane assay led to a profound reduction of vascularization, similar to anti-angiogenic drug sunitinib. Local administration of miR-7 in an *in vivo* murine neuroblastoma tumor model significantly inhibited angiogenesis and tumor growth. Finally, systemic administration of miR-7 using a novel integrin-targeted biodegradable polymeric nanoparticles that targets both EC and tumor cells, strongly reduced angiogenesis and tumor proliferation in mice with human glioblastoma xenografts. Transcriptome analysis of miR-7 transfected EC in combination with *in silico* target prediction resulted in the identification of OGT as novel target gene of miR-7. Our study provides a comprehensive validation of miR-7 as novel anti-angiogenic therapeutic miRNA that can be systemically delivered to both EC and tumor cells and offers promise for miR-7 as novel anti-tumor therapeutic.

## INTRODUCTION

Angiogenesis, the de novo formation of blood vessels from pre-existing vasculature, is not only a physiological process but also a driving factor in many pathologies [1]. Physiological angiogenesis is essential both during development and in adulthood, for example during wound healing and in the female reproductive system [2]. Angiogenesis occurs locally and is self-limited in time [3]. In contrast, pathological angiogenesis is often chronic and manifests in autoimmune diseases, age-related macular degeneration, atherosclerosis and cancer [3]. In many cancers angiogenesis is required for tumor progression: when the volume of a malignant mass becomes larger than several cubic millimeters, the increasing demand for nutrients and oxygen induces the production of angiogenic growth factors [4, 5]. Binding of these growth factors to receptors on nearby quiescent endothelial cells (EC) in pre-existing capillaries, leads to their activation, proliferation and ultimately formation of new vessels [6].

The critical role of angiogenesis in cancer progression has been validated clinically, and exploited therapeutically. Current anti-angiogenic therapeutics have shown clinical benefit for several cancer indications, but improvement in overall survival scores is minimal in many others [7, 8]. In addition, the development of resistance against anti-angiogenic single pathway therapies allows tumor re-growth due to activation of alternative pathways and remains a significant challenge for the treatment of cancer [9].

miRNAs are naturally occurring non-coding small double stranded RNA molecules of approximately 22 nucleotides in length, which are now established as important regulatory factors of gene expression in multiple pathways [10]. Recently their endogenous role has been shown to apply to angiogenesis [11, 12]. The first miRNA involved in angiogenesis was identified by Wurdinger et al. [13] and many others followed [14-19]. The ability of miRNAs to regulate multiple genes underlying angiogenesis provides exciting new opportunities for targeted anti-angiogenic therapy. Uniquely, miRNAs may combine broad anti-angiogenic activity against multiple targets with a presumed attractive toxicity profile since these miRNAs are also naturally expressed in normal tissue.

In the present study, a human miRNA lentiviral-based expression library encoding novel and known miRNAs [20] was screened in EC. miRNAs that inhibit proliferation of human primary EC and immortalized vascular EC were identified. The most potent miRNA, miR-7, was validated for anti-angiogenic activity *in vitro*. This miRNA was further validated *in vivo* with a chorioallantoic membrane (CAM) assay and a subcutaneous murine tumor model using local administration and electroporation. With strong support for its potential as an anti-angiogenic therapeutic agent, a clinically viable formulation which is based on a novel integrin targeted polymer-biodegradable nanoparticles delivery system, was used for intravenous administration. Delivery of miR-7 using this novel formulation demonstrated inhibition of tumor growth in a human glioblastoma xenograft model.

## RESULTS

### Identification of anti-angiogenic miRNA using a lentiviral based miRNA library

We aimed to identify miRNAs with a regulatory role in angiogenesis by screening a lentivirus-based expression library of 1120 human miRNAs. Viability of primary (HUVEC) and immortalized EC (EC-RF24) was assessed in a primary high throughput screen after infection of the cells. Initially, we identified 110 candidate miRNAs with either inhibitory or stimulatory effect on endothelial cell (EC) growth, of which 41 were confirmed in a secondary screen (Supplementary Fig. S1 and Table S1 for more details). In general the anti- and pro-proliferative activity of the lentivirus-expressed miRNAs was more pronounced in HUVEC than in EC-RF24 cells. In this study we focused on inhibitory miRNAs because the number of inhibitory hits was larger and the efficacy of the inhibitory hits on cell viability was larger than with stimulatory hits (see Table S1). To further narrow down to the most potent inhibitory miRNAs, our final selection consisted of miRNAs with >35% inhibitory effect in HUVEC (Table 1). Among the 6 selected miRNAs, hsa-miR-7-3 demonstrated the strongest anti-proliferative effect. The sequence of the hsa-miR-7-3 lentivirus was confirmed by Sanger sequencing. Stem-Loop RT-PCR showed that the pre-miRNA-7 hairpin is processed into mature miR-7 (hsa-miR-7-5p, Supplementary Table S2). We therefore selected miR-7 for further validation as an anti-angiogenic miRNA candidate.

**Table 1:** Final list of six endothelial anti-proliferative pre-miRNA from the lentiviral library in HUVEC and EC-RF24 Results are shown as % of viable cells compared to Empty Vector controls using MTS-read-out. (See Supplementary Fig. S1 and Table S1 for more detail)

	% of viability (MOI 100)		% of viability (MOI 200)	
Lentivirus	HUVEC	EC-RF24	HUVEC	EC-RF24
hsa-miR-7-3	45	53	41	43
hsa-miR-142	52	88	46	81
hsa-miR-26b	60	72	53	57
hsa-miR-574	61	93	60	98
hsa-miR-9-2	61	85	56	65
hsa-miR-190b	63	75	58	72

### Anti-angiogenic activity of miR-7 mimic *in vitro*

**Figure 1:**
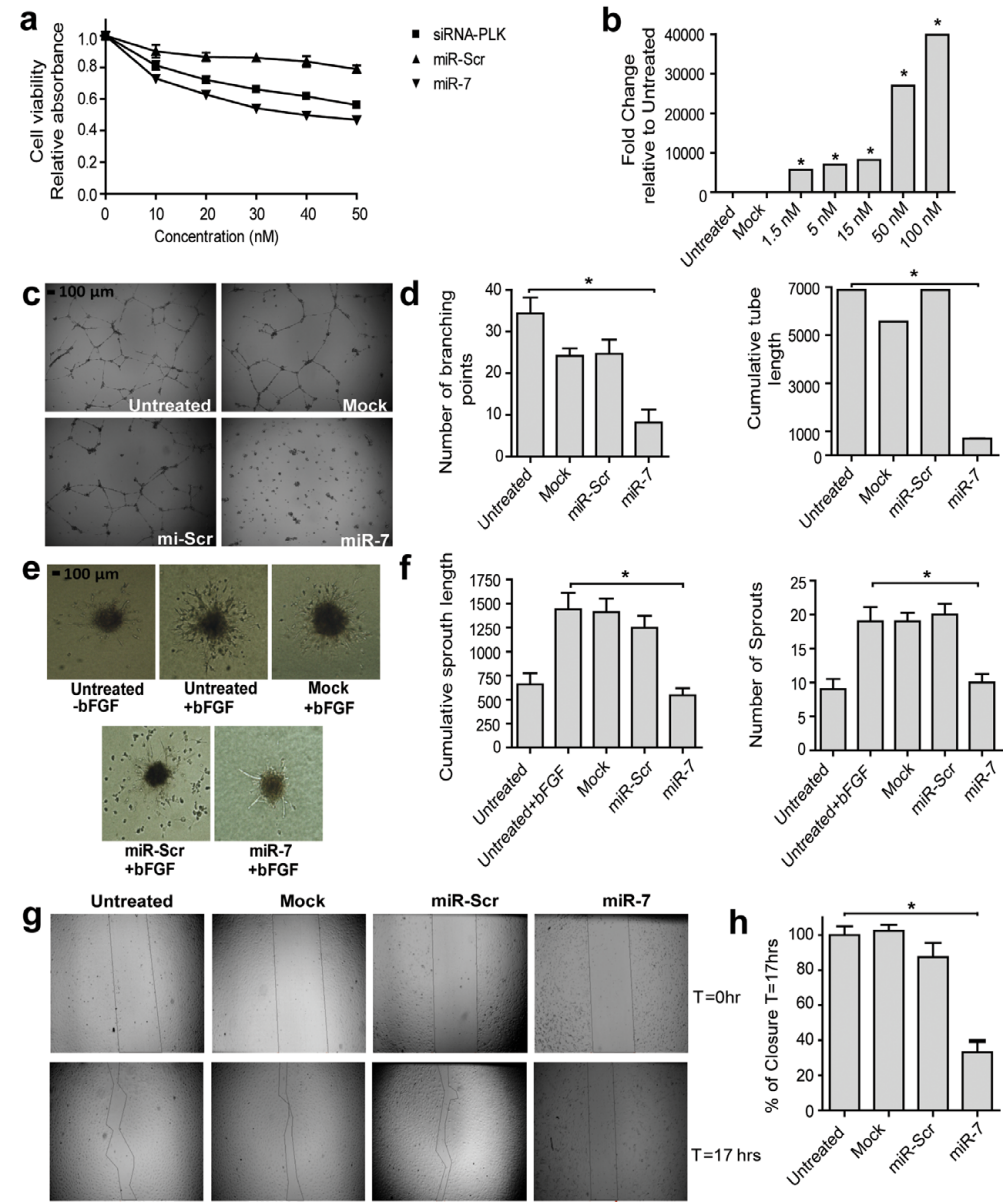
Anti-angiogenic property of miR-7 *in vitro* (a) *miR-7 inhibits HUVEC cell viability*. Cell viability was measured at 72 hrs after transfection using MTS-read-out. Cells were transfected with increasing concentrations of miR-7, miR-scr or siPLK-1. siPLK-1 and miR-scr were used as positive and negative control. Data are normalized to cell viability of untreated cells and plotted as mean values ± s.d. (n=3). (b) *Cellular miR-7 expression increases after transfection of HUVEC with increasing concentrations of miR-7*. miR-7 expression was measured by stem-loop RT-PCR at 72 hrs after transfection. Data are normalized to untreated cells and plotted as mean values ± s.d. (n=3), * *p<0.001*. (c-d) *miR-7 inhibits two-dimensional tube formation*. HUVEC were transfected with 50 nM miR-7 or miR-scr and seeded on matrigel at 48hrs after transfection. Pictures were taken at 17 hrs after seeding (magnification in Supplementary Fig. S11). Two-dimensional tube-formation was quantified by counting number of branching points and calculating the cumulative length of the tube of each image. Data are plotted as mean values ± s.d. (n= 3), * p<0.0001. (e-f) *miR-7 inhibits three-dimensional sprouting*. HUVEC were transfected with 50 nM miR-7 or miR-scr. 24hrs after transfection cell-spheroids were embedded in a collagen matrix in the presence of basic Fibroblast Growth Factor (bFGF). Untreated HUVEC in the absence of bFGF were used as negative control and the sprouting of miR-7 and miR-scr treated cells were compared to the sprouting of bFGF activated HUVEC. Pictures were taken at 16 hrs after embedding. Three-dimensional sprouting was quantified by counting the sprouts and calculating the cumulative length of 10 individual spheroids for each treatment. Data are plotted as mean values ± s.d. (n= 10), * p<0.0001. (g-h) *miR-7 inhibits migration*. HUVEC were transfected with 50 nM miR-7 or miR-scr. Cells were harvested at 48hrs after transfection and equal amount of cells were seeded in 24-well plate and wounded by a scratch. Images were taken right after the wound scratch (T=0) and at 17hrs after scratching (T=17) Wound closure was quantified by calculating unclosed surface area relative to surface area right after the scratch wound. Data are plotted as mean values ± s.d. (n=3),* p<0.001.

The anti-angiogenic activity of miR-7 was first studied by testing the anti-proliferative activity of a synthetic mimic of miR-7 in HUVEC at different concentrations (Fig. 1a). After transfection of HUVEC with miR-7 mimic cell proliferation was inhibited in a concentration dependent manner, up to 50% inhibition compared to a negative control miRNA with a scrambled sequence (miR-Scr). To confirm that transfection of HUVEC with increasing concentrations of miR-7 mimic led to increasing intra-cellular levels of miR-7, intra-cellular levels of miR-7 were measured by stem loop RT-PCR (Fig. 1b). The anti-proliferative effect of miR-7 was comparable to that of a positive control, i.e. siRNA against Polo-like kinase 1 (Plk1), a cell proliferation kinase. The anti-proliferative activity of miR-7 was confirmed in a BrdU cell proliferation assay (Supplementary Fig. S2a). Caspase-3 cleavage in HUVEC treated with miR-7 suggests that part of the anti-proliferative activity results in cell apoptosis (Supplementary Fig. S2b). To investigate whether miR-7 also affects EC tube formation and sprouting, HUVEC were transfected with miR-7 mimic, seeded on Matrigel at low density, and the formation of tubules in presence of growth factors was characterized (Fig. 1c). The results showed a statistically significant reduction in the number of branching points and cumulative tube length compared to the negative controls by approximately 76% and 90%, respectively (Fig. 1d). Similarly, a three-dimensional collagen based spheroid assay showed that transfection of EC with the miR-7 mimic resulted in a statistically significant reduction in the number of sprouts and cumulative sprout length of EC by approximately 47% and 62%, respectively (Fig. 1e and f). Since EC migration also plays an important role in angiogenesis, the effect of miR-7 mimic on wound closure was studied in an EC scratch assay (Fig. 1g). Indeed, miR-7 inhibited wound closure while miR-Scr transfected cells did not (Fig. 1h).

### Gene modulation by miR-7 mimic – HUVEC culture

**Figure 2:**
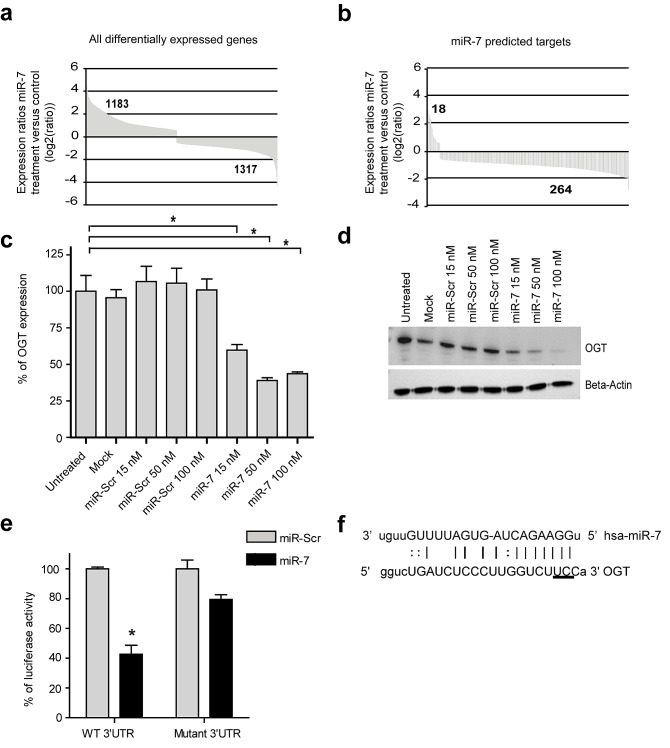
miR-7 modifies endothelial gene expression (a) *miR-7 changes the expression of 2500 HUVEC genes after transfection*. HUVEC were transfected with either miR-7 or miR-Scr and the transcriptome was quantified by RNA-Seq. All differentially expressed genes, which are statistically significant down- or upregulated compared to miR-Scr (p-value< 0.05) are plotted. (b) *Majority of miR-7 predicted target genes are downregulated*. Differentially expressed genes that are regulated by miR-7 and are a predicted target genes of miR-7 are plotted. (c) *mRNA expression of OGT is downregulated after miR-7 transfection*. HUVEC were transfected with increasing concentrations of miR-7 or miR-Scr. OGT expression was measured by RT-PCR at 48 hrs after transfection. Data are normalized to untreated cells and plotted as mean values ± s.d. (n=3), *p<0.001. (d) *OGT protein is downregulated after miR-7 transfection*. HUVEC were transfected with increasing concentration of miR-7 or miR-Scr. OGT expression was determined by Western Blot analysis at 48 hrs after transfection. Beta-Actin was used as an internal control. (e) *OGT is a target gene of miR-7*. Luciferase activities in Hela cells co-transfected with a luciferase- OGT 3′UTR plasmid containing either wildtype (WT) 3′UTR or mutated sequence and either miR-Scr or miR-7 at 24 hrs post transfection. Data are normalized to miR-Scr and plotted as mean values ± s.d. (n=3), *p<0.0002. (f) Alignment of miR-7 and OGT sequence. Mutations were generated on the potential target sequence (horizontally underlined).

One of the hallmarks of miRNAs is their ability to target and regulate multiple genes. To explain the anti-angiogenic properties of miR-7, gene expression analysis was performed using RNA-seq. Differential expression analysis showed that 2500 genes were significantly up- or downregulated after transfection of HUVEC with miR-7 mimic (*p*-value < 0.05, compared to miR-Scr, Fig. 2a). The majority, 1317, of the miR-7 modulated genes were down-regulated while 1183 were upregulated. Comparison of the list of down-regulated genes with a list of 11 clinically explored molecular targets in angiogenesis (Table 2) showed that eight genes matched the clinical target list. For each of these “therapeutic targets”, compounds are presently in clinical development (Table 2). The complete gene expression profile was analyzed using Ingenuity Pathway Analysis software (IPA). First, the set of regulated genes was overlaid with the 1051 predicted miR-7 targets, based on the TargetScan prediction tool in IPA. In total 282 predicted miR-7 targets were up- or downregulated within the set of 2500 genes (Fig. 2b). Consistent with the mechanism that miRNA down-regulate mRNA expression, 264 predicted targets (94%) were downregulated while only 18 (6%) were upregulated.

**Table 2:** miR-7 mediated differential expression of genes that are currently clinically explored as anti-angiogenic drug targets The log2(ratio) was calculated as described in Material and Methods and reflects the differential expression of genes in HUVEC treated with miR-7 and gene expression in HUVEC treated with miR-Scr. Eight out of 11 genes that are associated to novel anti-angiogenic drugs are down regulated

Genes highly involved in tumor angiogenesis	Expression ratio after miR-7 treatment versus control (log2(ratio))	Example of drug in clinical trial
VEGF-B	1.443	Aflibercept (Regeneron)
VEGF-C	−0.778	VGX-100 (Circadian)
Angiopoeitin-2	−0.805	PF-04856884 (Pfizer)
PDGF-D	0.899	CR-002 (CuraGen)
Jagged-1	−0.739	RO4929097 (Roche)
ADAM-10	−0.870	INCB3619 (InCyte)
FGF-2	−1.732	Gal-F2 (Galaxy/Roche)
CXCR4	1.169	BMS-936564 (Bristoll Myers Squibb)
S1PR1	−1.108	Fingolimod (Novartis)
S1PR3	−1.035	Fingolimod (Novartis)
NRP1	−0.801	MNRP1685A (Genentech) antibody

IPA was also used to identify biological processes that are regulated by miR-7 (Supplementary Table S3). Genes involved in functions associated with cell death and apoptosis were increased, while genes involved in functions such as cell migration, proliferation and vasculogenesis were decreased. Overlay of strongly down-regulated angiogenesis-associated genes (log2 ratio (miR-7 vs. miR-Scr), *p-*value <0.05) with predicted miR-7 targets (Supplementary Table S4) pointed to O-linked β-N-acetylglucosamine transferase (OGT) as potentially important mediator of miR-7 action. OGT is an enzyme that adds O-linked β-N-acetylglucosamine (*O*-GlcNAc) moieties to various nuclear and cytosolic proteins and gained interest as anti-tumor therapeutic target in recent years [21, 22]. The observed downregulation of OGT in miR-7 transfected HUVEC was confirmed by RT-PCR and Western Blot analysis over a concentration range of 15-100 nM (Fig. 2c and d). To proof that OGT is a direct target gene of miR-7, we measured luciferase activity in Hela cells transfected with a plasmid containing the 3′UTR sequence of OGT. The decrease in luciferase activity in the presence of miR-7 indicates a direct interaction between miR-7 and the 3′UTR of OGT (Fig. 2e). Mutagenesis of the 3′UTR sequence of the predicted binding site of miR-7 (Fig. 2f) restored luciferase activity, thereby confirming the specificity of the interaction between miR-7 and the OGT 3′UTR (Fig. 2e). To study the effect of OGT on angiogenesis, RNA interference was used to inhibit OGT expression in EC. As additional positive control Alloxan was used, which is a well known small molecule OGT inhibitor [23]. Both siRNA against OGT and Alloxan did not inhibit EC migration or EC tube formation *in vitro*, suggesting that OGT is a good marker gene of miR-7, but not the prime miR-7 target through which EC cell migration and tube formation are inhibited (Supplementary Fig. S3 and S4).

### Anti-angiogenic activity of miR-7 mimic *in vivo*– chick CAM assay

**Figure 3:**
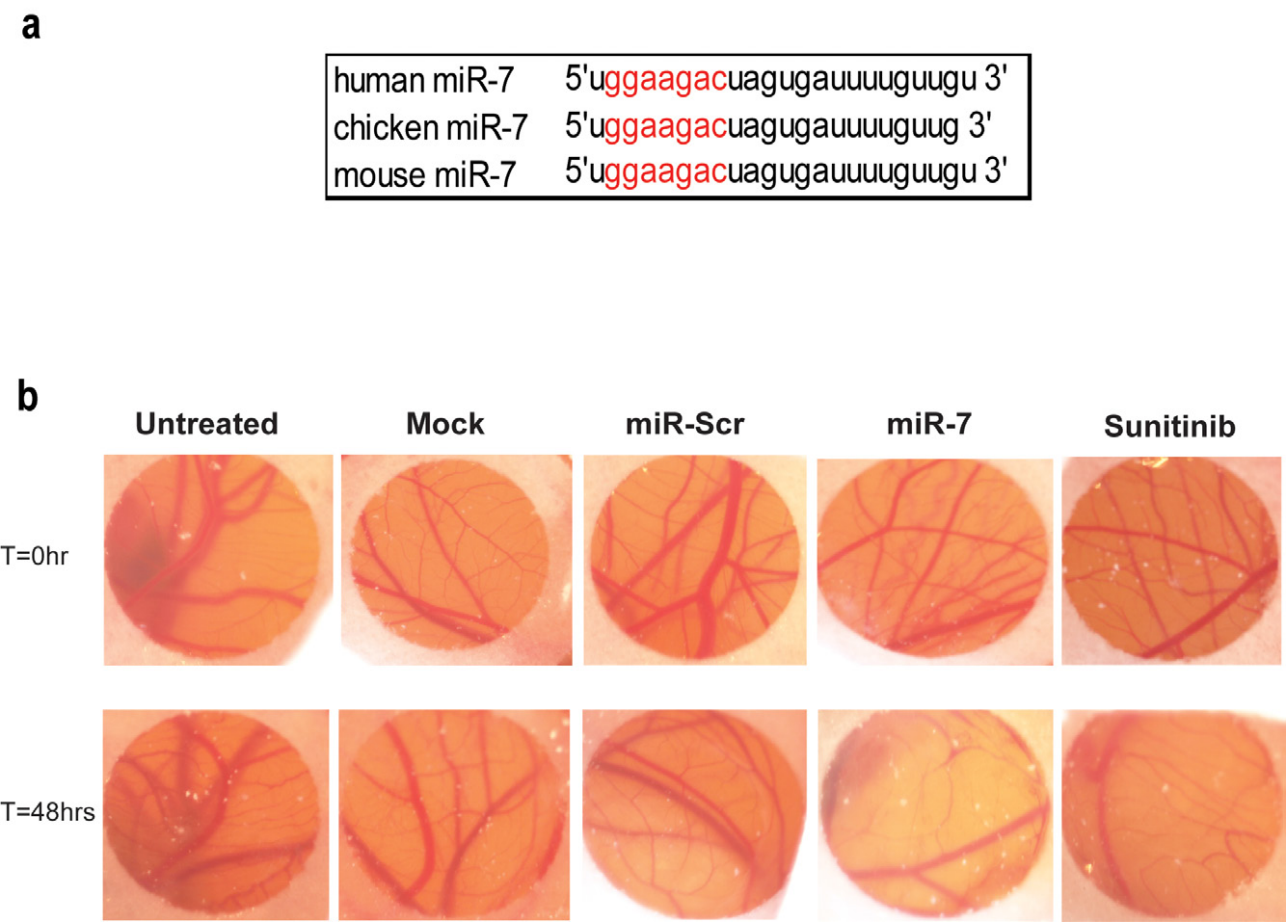
Effect of miR-7 on the CAM-assay (a) *Seed sequence of miR-7*. Illustration of conserved seed sequence of miR-7 among different species. (b) *miR-7 acts as vascular disrupting agent on CAM*. Chick CAMs were treated locally within a nitrocellulose ring with 300 picomol miR-7 or miR-Scr using Lipofectamine 2000 or with 200 picomol sunitinib. Untreated and mock treated CAM were used as controls. Representative photographs were taken prior to transfection (T=0) and at 48 hrs after transfection (T=48).

Mature miR-7 is conserved between chicken, human, and mouse (Fig. 3a). This streamlines translation of our *in vitro* data to *in vivo* tests for anti-angiogenic activity, starting with local treatment in a chick chorioallantoic membrane (CAM) assay (Fig. 3b). A reduction in vascular density in the regions between large blood vessels was visible in CAM treated with miR-7 mimic while vascular density was not reduced in untreated or miR-Scr treated CAM (Fig. 3b). This is indicative of a strong anti-angiogenic activity of miR-7. This was supported by the observation that treatment of CAM with a clinically approved multikinase anti-angiogenic drug, sunitinib, showed a similar inhibitory effect on vascularization.

### Anti-angiogenic activity of miR-7 mimic *in vivo*– local tumor administration

**Figure 4:**
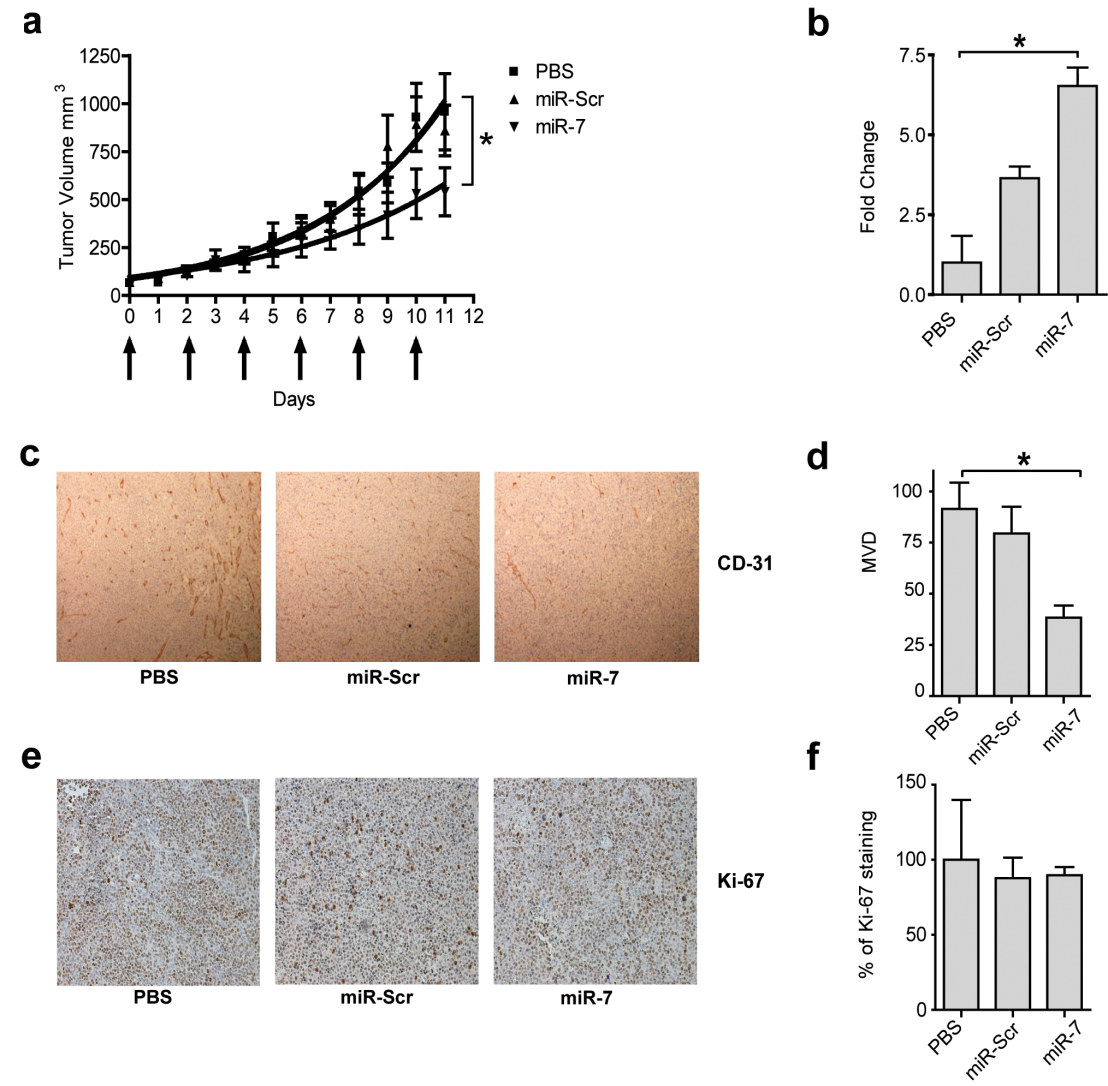
Inhibitory effect of miR-7 on tumor growth by local delivery (a) *miR-7 inhibits tumor growth after local delivery*. AJ mice bearing tumors with Neuro2A cells were treated locally with 10 μg miR-7 or 10 μg miR-Scr or PBS by intratumoral injection and electroporation. Arrows below the graph indicate treatment schedule (6 treatments, every other day). Data are plotted as mean values ± SEM (n=7), *p<0.05*. (b) *Increased presence of miR-7 in miR-7 treated animals*. The day after the last injection tumors were removed and total RNA was isolated. Delivery of miR-7 by electroporation into the tumor tissue was determined by stem loop RT-PCR. Values were normalized to U6 expression in the tumors. Data are plotted as mean values ± s.d. (n=3), *p<0.001. (c-d) *miR-7 reduces angiogenesis in vivo after local delivery*. Tumor sections were stained for CD31 (in brown) and microvessel density (MVD) was quantified by counting blood vessels in 6 random high magnification fields in each sample. Data are plotted as mean values ± s.d. (n=4), *p< 0.01. (e-f) *miR-7 does not affect proliferation in Neuro2A tumors*. The effect of miR-7 on tumor cell proliferation was determined by Ki-67 staining (in brown). Quantification of the images was performed in the same way as described in (c-d), and expressed as percentage of the PBS treated group. Data are plotted as mean values ± s.d. (n=4). Magnification of Fig 4c and 4e in Supplementary Fig. S12.

Based on the potent anti-angiogenic activity of miR-7 observed both *in vitro* and *in vivo* in the CAM assay, the anti-angiogenic potency and inhibitory effect on tumor growth was investigated in a subcutaneous neuroblastoma (N2A) mouse tumor model using intratumoral injections and electroporation. The miR-7 mimic (10 μg) treated mice exhibited a 43% reduction in tumor growth compared to both the PBS and miR-Scr negative control treated mice (Fig. 4a). Stem-loop RT-PCR was used to determine the relative tumor amounts of miR-7 in the different treatment groups. Tumors of miR-7 treated animals showed significantly higher miR-7 levels compared to the control groups (Fig. 4b). The biochemical process underlying tumor growth inhibition by miR-7 mimics was investigated using immunohistochemical (IHC) detection of CD31, an endothelial cell marker for microvessel density (Fig. 4c). MiR-7 mimic treated tumors displayed a reduced microvessel density, indicative of anti-angiogenic activity of the treatment (Fig. 4d). However, no differences in expression of Ki-67, a marker for proliferation, were detected among the treatment groups (Fig. 4e and f). These data suggest that inhibition of angiogenesis is the prime mechanism for the N2A tumor growth suppression upon intratumoral delivery of miR-7. The lack of efficacy on tumor cell proliferation *in vivo* is corroborated by the observation that miR-7 did not inhibit cell viability of N2A cells *in vitro* (Supplementary Fig. S5).

### Anti-angiogenic activity of miR-7 mimic *in vivo*– systemic tumor neovasculature targeting

**Figure 5:**
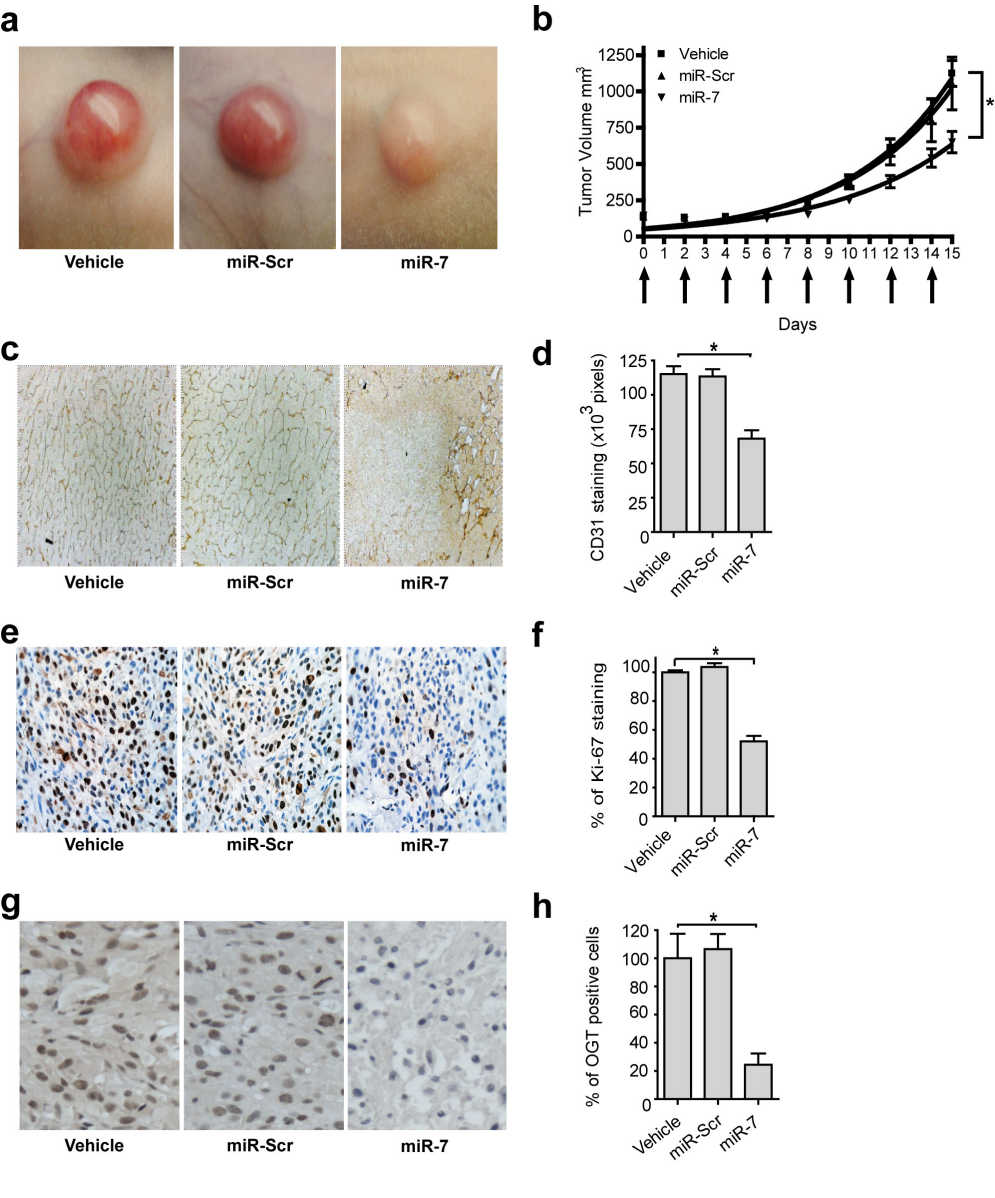
Inhibitory effect of miR-7 on tumor growth by systemic delivery (a) *miR-7 treated animals show pale and less vascularized tumors*. Macroscopic images of the tumors after the 7^th^ dose administration. (b) *miR-7 inhibits tumor growth after systemic delivery*. Athymic Nude-Foxn1^nu^ mice bearing U-87 MG tumors were injected intravenously with αvβ3/αvβ5 targeted miR-7 nanoparticles (3 mg/kg miRNA). Arrows in the graph indicate the days of treatment (8 treatments, every other day). miR-7 treated mice showed significant tumor growth inhibition compared to vehicle treated mice. Data are plotted as mean values ± SEM (n=10), *p<0.05. (c-d) *miR-7 reduces angiogenesis after systemic delivery*. Tumor sections were stained for CD31 (in brown) and quantified by counting CD31 positive staining area (pixels) in 6 random fields in each tumor. Data are plotted as mean values ± s.d. (n=5), *p<0.001. Magnification of Fig 5c in Supplementary Fig. S13. (e-f) *miR-7 reduces cell-proliferation in U-87 MG tumors after systemic delivery*. Anti-proliferative effect of systemically delivered miR-7 was determined by Ki-67 staining, indicated as brown spots. Quantification of Ki-67 was performed by counting of the stains in 6 random fields in each tumor section and proliferation was expressed as percentage of PBS treated mice. Data are plotted as mean values ± s.d. (n=5), *p<0.001. (g-h) *miR-7 reduces OGT in U-87 MG tumors after systemic delivery*. Tumor sections stained for OGT (brown nuclei and brownish cytoplasm) and quantified by counting of the stains in 6 random fields in each tumor section. Data are plotted as mean values ±s.d. (n=5), * *p=0.0004*.

Clinical application of miRNA-based therapeutics is dependent on systemic administration and intracellular delivery of the miRNA (mimic) to the target site. Studies have shown an ability of cRGD-targeted nanoparticles to provide neovasculature targeting and VEGF pathway siRNA inhibition of angiogenesis in murine models [24-26]. Therefore, this approach was selected for systemic delivery of miR-7. Hereto, a novel biodegradable neovasculature targeted nanoparticles formulation was developed and used to investigate the anti-tumor activity of miR-7 following intravenous administration in human glioblastoma U-87 MG bearing mice. U-87 MG tumor bearing mice were treated with miR-7 mimic using a cyclic Arginine-Glycine-Aspartic acid (cRGD) peptide coupled biodegradable polyamide nanoparticles, targeting integrins αvβ3 and αvβ5. These integrins are present on tumor EC neovasculature, as well as on certain tumor cells [27], such as observed with the U-87 MG tumor cell line (Supplementary Fig. S6). The results demonstrate that targeted systemic delivery of miR-7 inhibited tumor angiogenesis and growth. Tumors of the miR-7 treatment group were pale and less vascularized compared to those in control groups (Fig. 5a) and systemic delivery of miR-7 mimic inhibited tumor growth by 42% after two weeks of treatment (Fig. 5b). The anti-angiogenic property of miR-7 was also observed microscopically with a statistically significant reduction of immunohistochemical staining of CD31 in tumor tissue from the miR-7 treated mice (Fig. 5c and d). Moreover, tumor tissue from the miR-7 treated mice contained considerable amounts of necrotic lesions, which is an indication of hypoxia from reduced angiogenesis. Systemic delivery of miR-7 mimic not only reduced tumor angiogenesis but also reduced tumor proliferation as demonstrated by the statistically significant reduction in Ki-67 staining of miR-7 treated tumor tissue (Fig. 5e and f). This was confirmed by *in vitro* studies that demonstrated reduced proliferation of U-87 MG cells upon transfection with miR-7 mimic (Supplementary Fig. S7). To confirm effective delivery of miR-7 into the tumor tissue we performed IHC staining of OGT, one of the target genes of miR-7 (Fig. 5g). miR-7 treated mice showed a statistically significant reduction in OGT levels (Fig. 5h). Downregulation of OGT by miR-7 is not EC specific, but was also confirmed in U-87 MG cells *in vitro* (Supplementary Fig. S8). The observed inhibition of tumor growth can thus be ascribed to a combination of an anti-angiogenic effect of miR-7 delivered to tumor-associated EC and an anti-proliferative effect of miR-7 delivered to tumor cells. Similar to EC, downregulation of OGT in U-87 MG cells by RNAi interference did not inhibit cell viability (Supplementary Fig. S9), indicating that also in tumor cells OGT is a good marker for miR-7 delivery but not for miR-7 efficacy.

## DISCUSSION

This study has identified miR-7 as a prominent regulator of angiogenesis. In a functional screen with a lentiviral miRNA library miR-7 was identified as inhibitor of EC proliferation. The anti-angiogenic property of lentivirally expressed miR-7 was confirmed by investigating the efficacy of miR-7 synthetic mimics in multiple *in vitro* angiogenesis assays, each focusing on different hallmarks of angiogenesis. MiR-7 not only inhibited EC proliferation by 50%, but also inhibited migration of EC by nearly 70%. In more complex *in vitro* angiogenesis assays, miR-7 inhibited the ability of HUVEC to form two-dimensional tubules on matrigel and three-dimensional sprouts in collagen. Together, these studies demonstrate strong anti-angiogenic activity of miR-7 upon overexpression in EC. In earlier studies, miR-7 was not picked up as miRNA involved in angiogenesis in an EC specific differential expression screen (13), which may suggest that miR-7 has no physiological role in EC to regulate angiogenesis.

Apart from the here described newly discovered anti-angiogenic effect, human miR-7 was first described by Lim et al. in 2003 [28] and later this miRNA was found to be associated with anti-tumorigenic effects in glioma, hepatocellular carcinoma cells, and head and neck cancer cells [29-34]. In these cells anti-tumorogenesis is modulated amongst others through direct targeting of both the PI3K/Akt pathway and EGFR [29-34]. Our observation that miR-7 inhibits proliferation of U-87 MG cells confirms the anti-tumorigenic effect of miR-7 in tumor cells.

To elucidate the mechanism of action by which miR-7 exerts the anti-angiogenic effects, transcriptional analysis of miR-7 mimic transfected HUVEC was performed. Pathway analysis of gene expression changes implicated, amongst others, suppression of neovascularization. Overlay of the miR-7 predicted target genes with down-regulated genes with a functional annotation in neovascularization led to selection of OGT as putative novel target of miR-7. OGT is an enzyme that is involved in the hexosamine biosynthetic pathway which adds an *O-*GlcNAc moiety to the free hydroxyl group of select serine and threonine residues on a diverse population of nuclear and cytosolic proteins [35, 36]. Downregulation of OGT in tumor cells leads to increased degradation of the oncogenic transcription factor Forkhead transcription factor (FoxM1) protein, a direct regulator of VEGFR-2 and FoxF1 expression in EC [21, 37]. Here we show for the first time that OGT is a target of miR-7, suggesting that the anti-angiogenic effect of miR-7 in EC can, at least partly, be mediated by downregulation of OGT. However, inhibition of OGT in EC does not inhibit EC tube formation or migration, which indicates that OGT is a suitable biomarker for miR-7 activity, but does not explain the anti-angiogenic activity of miR-7 in EC. It is noted that other miR-7 targets, such as EGFR and PI3K were not down-regulated in miR-7 treated EC based on the RNA-seq analysis (Supplementary Table S7), suggesting that the anti-proliferative activity of miR-7 occurs through other pathways in EC than in tumor cells. Investigation of gene expression levels of known miR-7 target shows that EGFR and PI3K and most of the other known miR-7 targets are not affected in HUVEC after miR-7 transfection (Supplementary Fig S10). The mechanism through which miR-7 regulates distinct pathways in EC and tumor cells will be subject of future research.

Not only did miR-7 inhibit growth factor induced angiogenesis *in vitro* but miR-7 also impaired developmental angiogenesis in chicken embryo CAM with potency comparable to sunitinib. In order to evaluate and validate the anti-angiogenic effect of miR-7 mimic *in vivo*, we used a Neuro2A syngeneic tumor model, because these tumors are well vascularized and respond well to anti-angiogenic therapy [25, 38, 39]. Tumor treatment with intratumoral injection of miRNA delivered by electroporation showed that the miR-7 mimic decreased angiogenesis and reduced tumor growth. Proliferative status of tumor cells in the different treatment groups was comparable, indicating that the primary mechanism of action for the inhibition of tumor growth in this model was inhibition of angiogenesis.

Electroporation has limited clinical application since it can only deliver miRNA locally in superficial tumors. For broader clinical application, a systemic delivery system is needed. We developed a novel αvβ3/αvβ5-integrin targeted nanoparticles for systemic delivery of miR-7 mimic to tumor EC and tumor cells and evaluated it in a human glioblastoma xenograft tumor model. Indirect evidence for systemic delivery of miR-7 to the tumor tissue was provided by the observation that mice treated with the miR-7 mimic-loaded, integrin-targeted nanoparticles had smaller, pale and less vascularized tumors than control mice. The nanoparticles developed in the current study might elicit dual activity, as not only the EC express high integrin levels, but also in tumor cells. This suggests that inhibition of miR-7 mediated tumor growth is caused by a combined effect on both tumor cells and tumor EC. Indeed, miR-7 inhibits proliferation of not only HUVEC, but also of U-87 MG cells *in vitro*. Furthermore, not only tumor vessel density was reduced, expression of the nuclear proliferation marker Ki-67 and expression of the newly identified miR-7 target gene OGT were also affected by miR-7 treatment *in vivo*.

The U-87 MG xenograft tumor model was selected because it is highly vascularized and responds well to anti-angiogenic therapy such as bevacizumab or sunitinib [40, 41], making it a suitable model to study the anti-angiogenic property of miR-7. Naturally, this model does not reflect the nature of glioblastomas in the clinic and recently it was shown that angiogenesis inhibition in glioblastoma patients may induce glioblastoma migration and invasion [42, 43]. In this work, the U-87MG model was used to explore the feasibility of miR-7 as anti-angiogenic agent and other therapeutically relevant tumor models will be explored in the near future.

The dual targeting of the cRGD-coated nanoparticles to both endothelial as well as cancer cells provides potential for high therapeutic efficacy of miR-7. Together with the well-known role of miR-7 as regulator of invasion and migration in cancer, the novel anti-angiogenic property of miR-7 and its regulation of diverse genes involved in angiogenesis strengthen its potential value as therapeutic agent for the treatment of cancer

## MATERIAL AND METHODS

### Cell Culture and reagents

Human Umbilical Vein Endothelial Cells (HUVEC) (Lonza) were cultured in EBM-2 medium (Lonza) supplemented with bullet kit (EGM-2, Lonza) containing several growth factors and 10% Fetal Calf Serum (FCS) (Sigma). Cells were used between passages 2 and 7. Immortalized EC (EC-RF24) (ABM#T0003) were cultured in M199 medium (Gibco) containing FCS (Sigma), Human Serum (HS) (Sigma), 100 U/ml Penicillin/Streptavidin (P/S) (Biochrom AG) and 200 nM L-glutamin (Sigma). Immortalized murine neuroblastoma (Neuro2A, ATCC CCL-131) were cultured in RPMI-1640 with 10% FCS and antibiotics (100 U/ml Penicillin, 100 μg/ml Streptomycin, and 0.25 μg/ml amphotericin B). Human glioblastoma U-87 MG cells (gift from Prof. James A. Mixson, University of Maryland, US) were cultured in DMEM containing 10% FCS.

### Stem-loop Reverse Transcription PCR

RNA isolation was perfomed using Trizol (Invitrogen) following the manufacturer′s protocol. The presence of miRNA was determined by Stem-Loop RT-PCR as described in Chen et. al. [44]. mRNA levels were determined according to standard protocol using home-made primers and SYBRGreen (Bio-Rad, CA, USA). For the stem-loop RT-PCR individual forwards primers were designed according to mature miRNA sequence in miRBase 16. The mRNA RT-PCR primers were obtained from PrimerBank [45]. As a housekeeping gene U6 was used for miRNA and HPRT, GAPDH and/or BGUS were used for mRNA. RT-PCR was performed on a BioRad CFX96 (CA,USA). Sequences of the primers are described in Supplementary Table S5.

### Proliferation assay with mature miRNA mimic

HUVEC, seeded in 96-well plate (7500 cells/well), were transfected with miRNA mimic using X-tremeGENE (Roche) on the following day according to manufacturer's protocol (0.5 μl X-tremeGENE for each 96-well). Mimics (Pre-miR™ miRNA Precursors, Ambion) and siRNA PLK-1 (ON-TARGET plus SMARTpool, Dharmacon) were tested at different concentrations. As negative control Pre-miR™ miRNA Precursor Negative Control #1 (Ambion) was used (miR-Scr). Cell viability was determined with the MTS assay according to manufacturer′s protocol (Promega).

### Tube formation

HUVEC, seeded in 6-well plate (8×10^4^ cells/well), were transfected with 50 nM miRNA mimic with Lipofectamine 2000 (Invitrogen, UK) on the following day according to manufacturer′s protocol in serum free medium. After 4 hrs, medium was replaced with EGM-2. After 48 hrs incubation, a 96-well plate was coated with 50 μl Matrigel^TM^ (BD Biosciences, San Jose, CA) and incubated for 30 min at 37°C. Transfected HUVEC were counted, prepared in a cell suspension in EGM-2 medium and seeded on top of the Matrigel^TM^ (7500 cells/well). After 17 hrs incubation wells were imaged with a Nikon TE2000 microscope at 10× magnification. Tubule formation was quantified by counting the number of branching points and measuring the total length of capillary tubes in at least three independent wells from the same condition using NIH ImageJ software.

### Sprouting assay

HUVEC, seeded in a 6-well plate (8×10^4^ cells/well), were transfected with 50 nM miRNA mimic using X-tremeGENE (see above). After 48 hrs cells were suspended in culture medium containing 20% (v/v) methocell at a final density of 4×10^4^ cells/ml to form cell spheroids. Spheroids were embedded in collagen gel containing 100ng/ml bFGF (Reliatech, Braunschweich, Germany) and allowed to sprout for 16 hrs. Phase-contrast images were captured using a Hitachi GiGE camera 1.4MB, linked to an inverted Leica microscope DMI3000. *In vitro* capillary sprouting was quantified by measuring the cumulative sprout length and the number of sprouts per spheroid using NIH ImageJ software.

### Migration assay

HUVEC were transfected using Lipofectamine 2000 (see above). 48 hrs after transfection cells were counted and seeded in 24-well plates (6×10^4^ cells/well). Two hours after cell seeding a scratch wound was made using a yellow pipette tip vertically across each well. Wells were washed and medium was refreshed to remove cell debris. Images of the wells were taken on a Nikon TE2000 microscope at T=0, 6 and 17 hrs.

### Deep sequencing

HUVEC were seeded in a 6-well plate (8×10^4^ cells/well) and transfected with 15 nM miRNA mimic using X-tremeGENE (see above). After 72 hrs, RNA was isolated using Trizol (Invitrogen) reagent according to manufacturer′s protocol. Purified total RNA concentration was measured using a Qbit®Fluormeter and 30 μg total RNA was used to create RNA-seq libraries. Isolation of mRNA was performed using MicroPoly(A) Purist kit (Ambion) and mRNA-ONLY kit (Epicenter). Samples were prepared for sequencing using the SOLiD™ Total RNA-Seq Kit (Applied Biosystem). Relative expression was calculated as the ratio of reads mapping to a gene in the miR-7 transfected sample and the reads mapping to a gene in the miR-Scr transfected sample.

### 3′-UTR luciferase assay

The 3′UTR sequences (Supplementary Table S6) were synthesized by IDT technologies, and cloned into psiCHECK^TM^-2 (Promega). Hela cells seeded in 24-well plated, were co-transfected with 10 nM miR-7 or miR-Scr and 100ng/well 3′UTR psiCHECK^TM^-2 construct using Lipofectamine 2000 (Invitrogen) according to manufacturers protocol. After 48 hrs, cells were lysed using Passive Lysis Buffer and luciferase activity was measured using the Dual-Luciferase Reporter Assay System (Promega) and Glomax-Multi Detection system (Promega) in 96-well format according to manufacturer′s protocol.

### Immunoblotting

HUVEC, seeded in a 6-well plate (8×10^4^ cells/well), were transfected at different concentrations of miR-7 using X-tremeGENE (see above). After 48 hrs cells were lysed in 200 μl radioimmunoprecipitation assay (RIPA) buffer (ThermoFisher) containing protease inhibitors (1×) and EDTA(1×) for 20 min on ice. Lysates were centrifuged at 4°C at 13,500 RCF for 15 min to remove the debris. Equal amounts of protein were run on SDS-PAGE gels and subsequently transferred to nitrocellulose membrane. Blots were incubated with primary antibodies OGT (1:1000. Cell Signaling) and Beta Actin (1:1000. Cell Signaling) followed by peroxidase-conjugated secondary antibody (Cell signaling). Bands were visualized with SuperSignal West Femto Chemiluminescent substrate (Pierce). Images were acquired with Gel Doc XRS imaging system with quantity One analysis software (Bio-Rad). All images shown are representative of 3 independent experiments.

### Chorio Allantoic Membrane (CAM)-Assay

Fertilized white leghorn chicken eggs were pre-incubated at 37°C with 45% humidity. After 2 days 2-3 ml albumin was removed from the egg in order to detach the CAM from the shell. On day 10 after making a window in the shell, sterile nitrocellulose rings were placed on top of the CAM. Subsequently, the rings were loaded with 300 picomol miR-Scr or miR-7 mimics complexed with Lipofectamine 2000 in 20 mM Hepes buffered glucose (pH 7,4). 200 picomol Sunitinib (Sequoia Research Products) was used as positive control. On day 10 and 12 CAMs were photographed in ovo with a Olypmus e40 camera.

### Neuroblastoma *in vivo* model: local delivery

The neuroblastoma (Neuro2A) tumor growth experiment was a randomized blinded study performed under approval of the University of Utrecht animal ethics committee. The protocol is adapted from Vader et. al [46]. Six to eight weeks old normal male A/J mice (Harlan) housed in environmentally controlled rooms, were injected subcutaneously in the right flank with 100 μl Neuro2A cells (1×10^7^ cells/ml). Tumor sizes were measured daily with digital calipers and tumor volume was calculated by using the following formula: (width^2^ × length) × 0.52. Treatment started with a tumor volume between 40-70 mm^3^ by intratumoral injection of 10 μg of miRNA every other day followed by electroporation at a setting of 200V/cm using an ECM 830 electroporator (BTX) set to deliver 2×2 pulses at perpendicular angles (n=7 per group) [47]. On day 11 mice were sacrificed and the tumors were excised.

### Nanoparticle preparation for systemic administration *in vivo*

AparnaBio′s cRGD-targeted biodegradable delivery system (cRGD-PPC) was used for systemic delivery of microRNA mimics *in vivo* [48]and Liu et al. (manuscript in preparation). miRNA/polymer nanoparticle were prepared at room temperature by dissolving miRNA in HGB (20 mM HEPES, 5% Glucose, pH 7.1). An equal volume of the polymer solution was mixed with miRNA solution (final concentration 0.2 mg/ml) to achieve a molar ratio of polymer nitrogen to RNA phosphate (N/P ratio) of 6. Mixed solutions were then briefly vortexed and left for equilibration for 30 min at room temperature to allow nanoparticle formation.

### Glioblastoma xenograft tumor *in vivo* model: systemic delivery

Systemic delivery of miR-7 nanoparticles in tumor bearing mice was a randomized, blinded study which was performed in an AALAC certified vivarium (Biomedical Research Institute, Rockville, MD, USA). Five to six weeks old female athymic mice (Athymic Nude-Foxn1^nu^ mice (Harlan, Indianapolis, USA) housed in environmentally controlled rooms, were inoculated with U-87 MG cells (5×10^6^ cells/ml suspended in 1:1 PBS/Matrigel) in their right flank. Tumor sizes were measured in the same way as described above. Mice were treated by intravenous injection every other day with the cRGD-PPC nanoparticles containing 60 μg miRNA mimic, or mir-Scr, (n=10 mice per group, 3 mg/kg). PBS was used as control. One day after the 8^th^ injection mice were sacrificed and the tumors were excised.

### Tissue analysis

Mouse tumors were formalin-fixed, paraffin embedded and 6 μm sections were cut for immunostaining. Sections were deparaffinized in xylene and rehydrated in a graded series of alcohol baths, blocked in 5% normal goat serum (Dako) and subsequently incubated either with rabbit anti-mouse CD31 antibody (1:500)(Santa Cruz), with rabbit anti-mouse Ki-67 antibody (1:300)(Abcam), or with rabbit anti-mouse OGT antibody (1:500)(Cell Signaling). Next, sections were incubated with 0.3% H_2_O_2_/PBS, followed by polyHRP goat anti-rabbit IgG incubation (Bright Vision). Finally, antibody binding was visualized with 3,3′-diaminobenzidine (DAB).

### Statistical Analysis

All statistical analyses were performed with Prism (GraphPad) or Excel (Microsoft). Two-tailed Student′s *t* test or One-Way ANOVA was used to calculate the statistical significance. A *p*-value <0.05 was considered to be statistically significant.

## SUPPLEMENTARY INFORMATION FIGURES AND TABLES


