# Supplementary material for: Systemic miRNA-7 delivery inhibits tumor angiogenesis and growth in murine xenograft glioblastoma
Source: Oncotarget. 2014 Jul 18;5(16):6687–700. doi: 10.18632/oncotarget.2235 (PMC4196156; doi:10.18632/oncotarget.2235)
Supplement: Supplementary file 1 [file oncotarget-05-6687-s001.pdf]

Systemic miRNA-7 delivery inhibits tumor angiogenesis and growth in murine xenograft glioblastoma

Supplementary Information

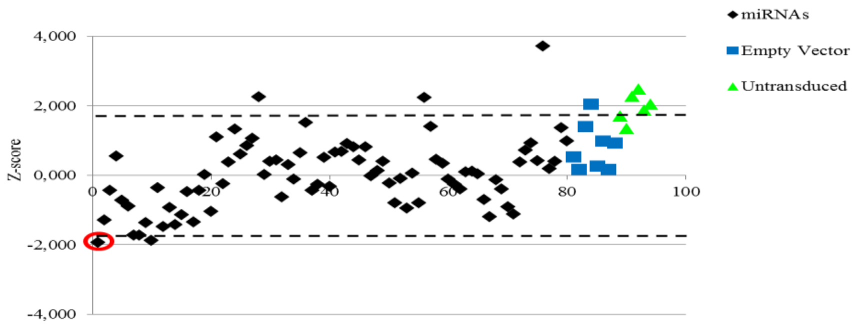

Figure S1: Z-score for each duplicate for each miRNA on one of the screening plates. This figure represents the Z-score calculated for each duplicate in the screen using HUVEC. The Z-score was calculated based on the MTS cell viability read-out for each of the 96 conditions in duplicate plates. The Z-score of for each miRNA is depicted by black diamonds. Untransduced and Empty Vector transduced controls are depicted by green triangles and blue squares, respectively. Diamond circled in red indicate miRNA-7. The construction of the lentiviral miRNA expression library is described by Poell et al. (18). The library was screened in duplicate, with a Multiplicity of Infection (MOI) threshold of 50 with HUVEC ($2x10^3$ cells/well) and EC-RF24 cells ($1.5x10^3$ cells/well). The cell viability of transduced cells was assessed 8 days after infection by using the MTS assay according to manufacturer's protocol (Promega). The duplicate MTS results were averaged and the Robust Z-score was calculated per plate. Using a cut-off Z-score of ≤ -1.75 or ≥ +1.75 and an average % cell viability of <85 or >115. The average cell viability was calculated per duplicate plate as % cell viability compared to empty vector controls. 110 miRNA hits were selected to be confirmed in a secondary screen using normalized titers (MOI 50) per miRNA. The secondary screen was performed using the same transduction protocol and MTS read-out in two cell lines. 41 out of 110 hits were confirmed.

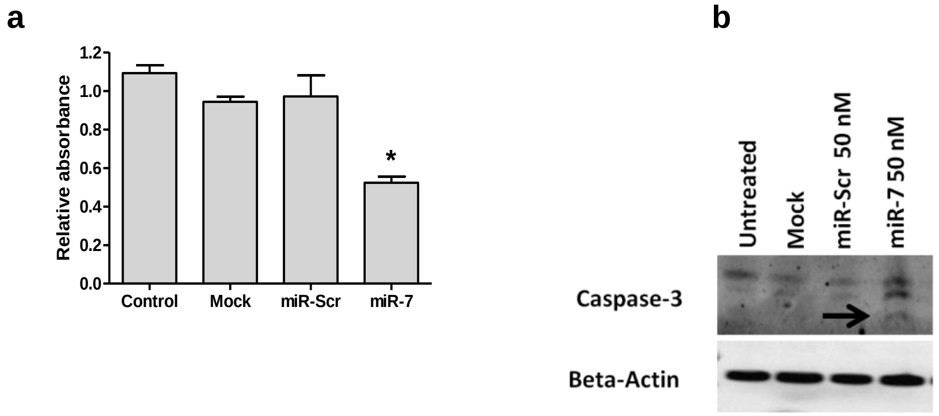

Figure S2: Anti-proliferative and apoptotic effect of miR-7 on HUVEC.(a) HUVEC, seeded in 96-well plate (5x10^4 cells/ml), were transfected with 50 nM miRNA-7 mimic or miR-Scr (Pre-miR™ miRNA Precursors, Ambion) using X-tremeGENE (Roche) on the following day according to manufacturers protocol (0,5 µl X-tremeGENE for each 96-well). Cell proliferation was determined with a BrdU assay kit according to manufacturer's protocol (Roche). Data are presented as mean values ± s.d. (n=4), *$p<0.0001$. **(b)** HUVEC, seeded in a 6-well plate (8x10^4 cells/well), were transfected with 50 nM miR-7 using X-tremeGENE (see above). After 48 hrs cells were lysed in 200 µl radioimmunoprecipitation assay (RIPA) buffer (ThermoFisher) containing protease inhibitors (1x) and EDTA (1x) for 20 min on ice. Lysates were centrifuged at 4°C at 13,500 RCF for 15 min to remove the debris. Equal amounts of protein were run on SDS-PAGE gels and subsequently transferred to nitrocellulose membrane. Blots were incubated with primary antibodies Caspase-3 (1:1000, Cell Signaling) and Beta Actin (1:1000, Cell Signaling) followed by peroxidase-conjugated secondary antibody (Cell signaling). Bands were visualized with SuperSignal West Femto Chemiluminescent substrate (Pierce). Arrow indicates cleaved capase-3 which appears in miR-7 treated samples.

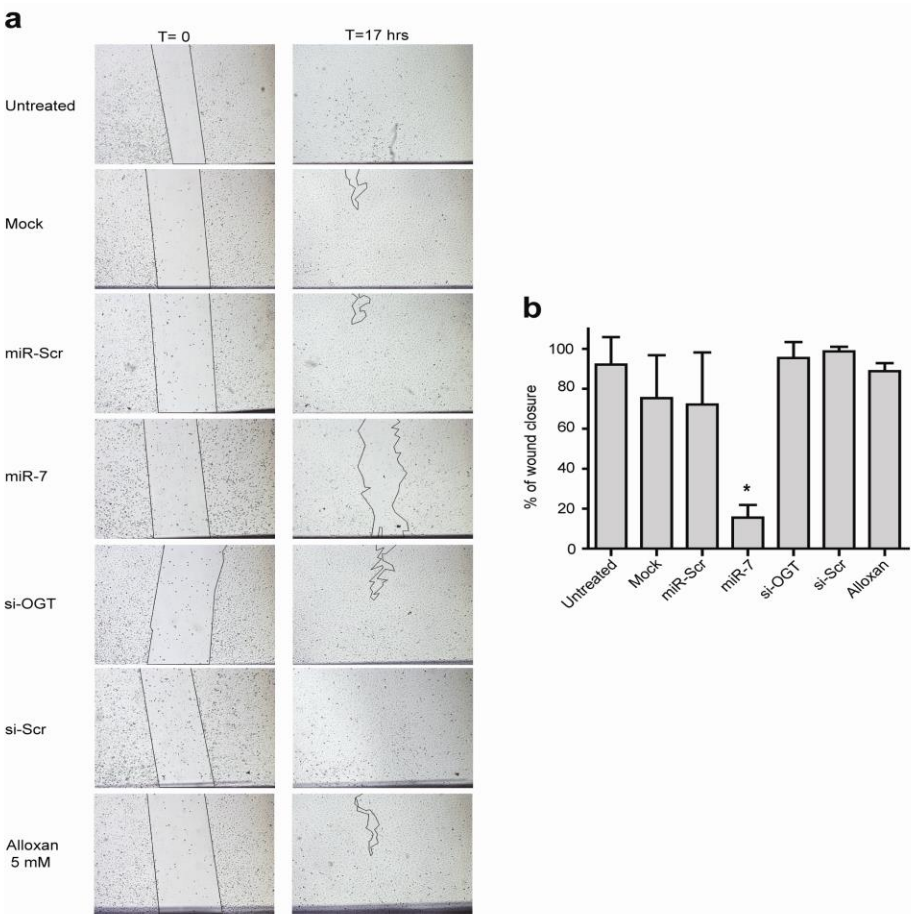

Figure S3: OGT is not involved in endothelial cell migration. (a) *miR-7 inhibits cell migration, whereas Alloxan and siRNA OGT do not.* HUVEC were transfected with 50 nM miR-7, miR-Scr, si-OGT, or si-Scr or incubated with 5 mM Alloxan (based on protocol adapted from Sakurai et al. Biol. Pharm. Bull. 2001, 24;876-882). Cells were harvested 48 hrs after treatment and equal amount of cells were seeded in a 24-well plate and wounded by a scratch. Images were taken right after the wound scratch (T=0) and at 17 hrs after scratching (T=17 hrs). **(b)** Wound closure was quantified by calculating unclosed surface area right after the scratch wound. Data are plotted as mean values ± s.d. (n=3),*$*p<0.001$*.

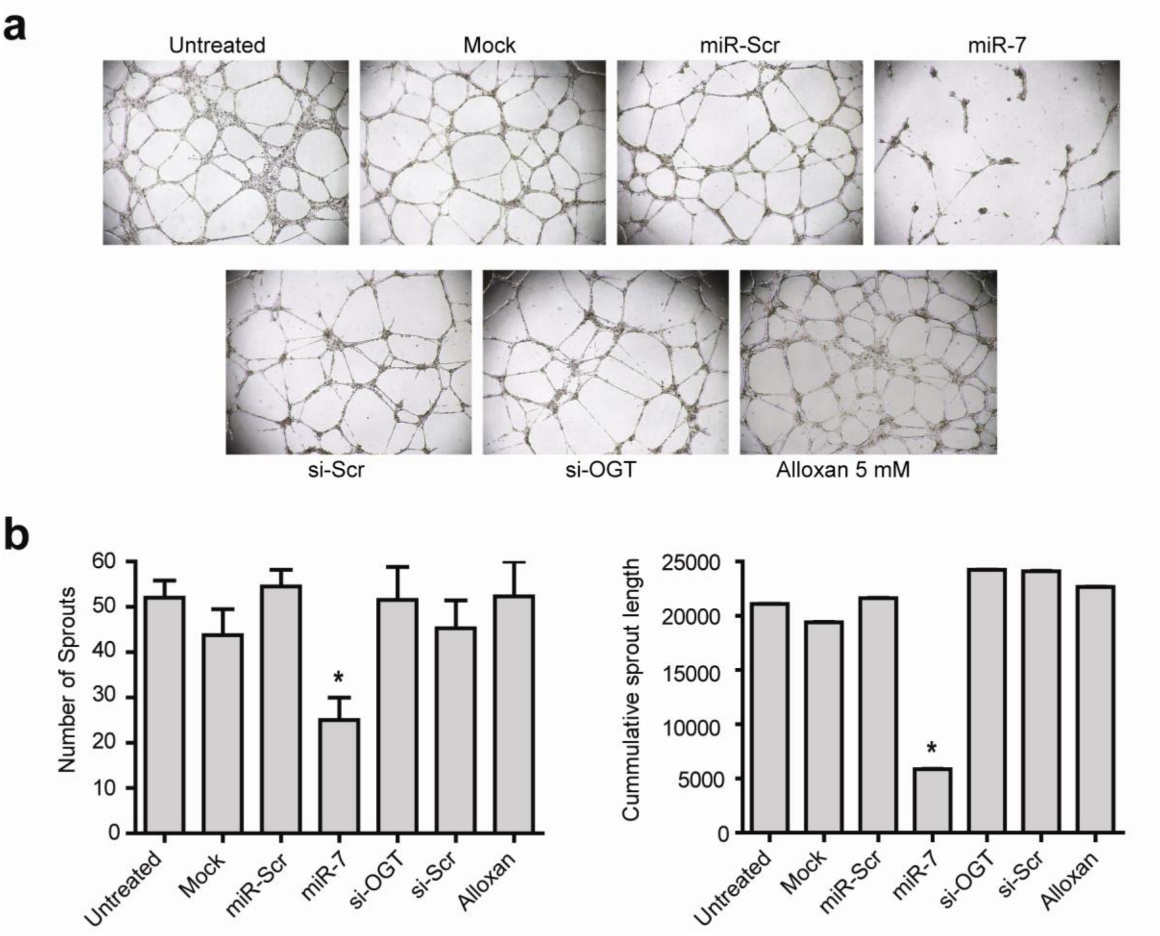

Figure S4: OGT is not involved in tube formation. (a) *miR-7 inhibits two-dimensional tube formation, whereas Alloxan and siRNA OGT do not*. HUVEC were transfected with 50 nM miR-7, miR-Scr, si-OGT, or si-Scr or incubated with 50 nM Alloxan (based on protocol adapted from Sakurai et al. Biol. Pharm. Bull. 2001, 24;876-882). Cells were harvested 48 hrs after treatment and equal amount of cells were seeded on matrigel. Images were taken at 17 hrs after seeding. **(b)** Two-dimensional tube-formation was quantified by counting number of branching points and calculating the cumulative length of the tube of each image. Data are plotted as mean values ± s.d. (n=3), **p<0.001*.

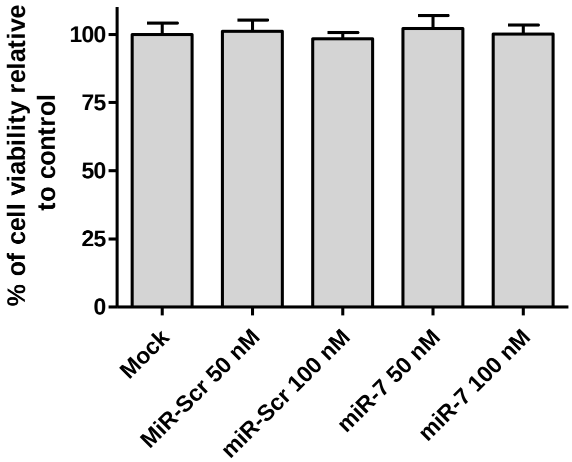

Figure S5: miR-7 does not inhibit N2A cell viability. N2A cells seeded in a 96-well plate ($5x10^3$ cells/well) were transfected were transfected with 50 or 100 nM miRNA-7 mimic (Pre-miR™ miRNA Precursors, Ambion) using X-tremeGENE (Roche) on the following day according to manufacturers protocol (0,5 µl X-tremeGENE for each 96-well). Cell viability was determined with a MTS assay kit according to manufacturer's protocol (Promega) at 72 hrs after transfection. Data are presented as mean absorbance values ± s.d. (n=4).

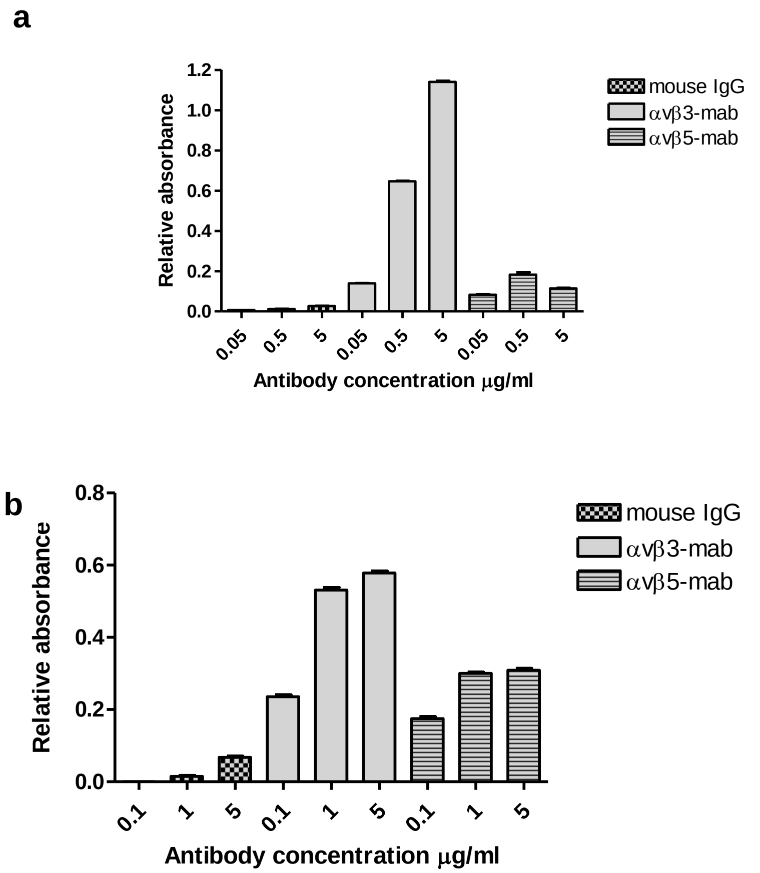

Figure S6: αvβ3 and αvβ5 expression in human U-87MG glioblastoma cells and HUVEC. Human U-87 MG glioblastoma cells ($1x10^5$ cells/ml) and HUVEC ($0.5x10^3$ cells/ml) in a 96-well plate were incubated with αvβ3 (EMD Millipore), αvβ5 (EMD Millipore), and mouse IgG1 monoclonal (Sigma) antibodies diluted in 3% BSA/PBS (with Mg^{2+} and Ca^{2+}) at different concentrations, for 1 hr at room temperature. Mouse IgG1 antibody was used as negative control. Next, cell were incubated with secondary HRP conjugated anti-mouse IgG antibodies (1:2000). The expression of the integrins was detected by adding TMB substrate allowing color formation and followed by stop solution ($N_2H_2SO_4$). Optical density was measured by 450 nm. U-87 MG **(a)** and HUVEC **(b)** incubated with monoclonal antibodies against mouse IgG1, αvβ3, and αvβ5 at different concentrations show dose dependent binding of antibodies to the cells indicating that cells express integrin αvβ3 and αvβ5. Data are mean values ± s.d. (n=4).

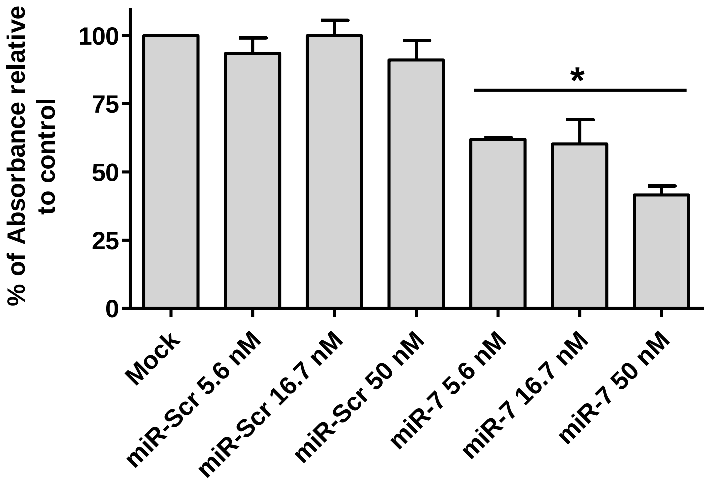

Figure S7: miR-7 inhibits U-87 MG cell viability. Viability of U-87 MG cells was measured with WST-1 assay at 96 hrs after transfection. Cells seeded in a 96-well plate ($3x10^3$ cells/well) were transfected with 5.6-50 nM miRNA with Lipofectamine RNAiMax Reagent (Invitrogen) according to manufacturer's protocol. Metabolic activity was measured 30 min after addition of 10 µl of WST-1 reagent (Roche Diagnostics) and reading absorbance at 450 nm in a microplate reader. miR-7 shows a dose dependent decrease in cell viability as represented by % of absorbance relative to control. Data are mean values ± s.d. (n=3), *$p<0.01$.

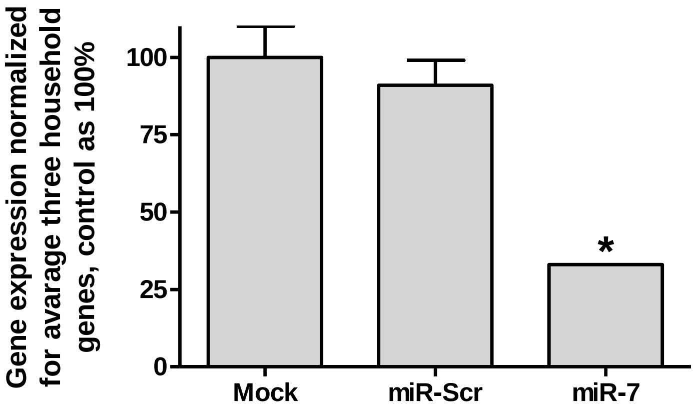

Figure S8; Modified OGT expression in U-87 MG upon miR-7 transfection.U-87 MG cells were transfected in the same way described in material & methods for RNA-seq analysis above. 72 hrs post tranfection RNA of the cells was isolated using Trizol according to manufacturer's protocol. RT-PCR of miR-7 target genes according to literature indicated in the manuscript. Gene expression was normalized for average three household genes (HPRT, GAPDH, GUSB).

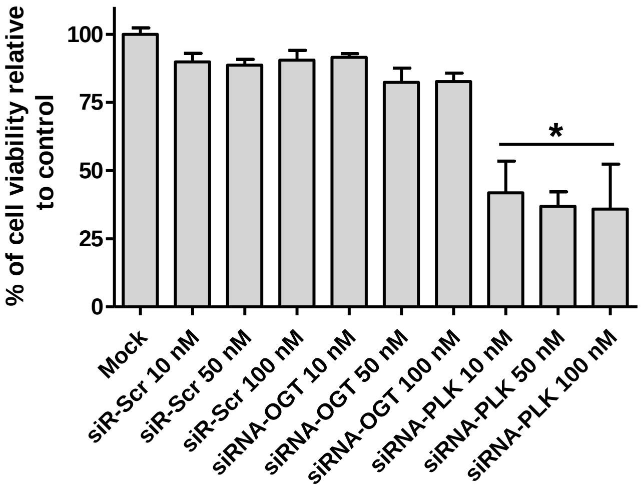

Figure S9: Si-OGT does not inhibit U-87 MG cell viability. U-87 MG cells seeded in a 96-well plate (5x10^3 cells/well) were transfected were transfected with 10, 50, 0r 100 nM siRNA against PLK or OGT using Lipofectamine RNAiMax Reagent (Invitrogen) according to manufacturer's protocol (0,375 µl Lipofectamine RNAiMax Reagent for each 96-well). siRNA-PLK was used as positive control. Cell viability was determined with a MTS assay kit according to manufacturer's protocol (Promega) at 72 hrs after transfection. Data are presented as mean absorbance values ± s.d. (n=4), *$p<0.0001$.

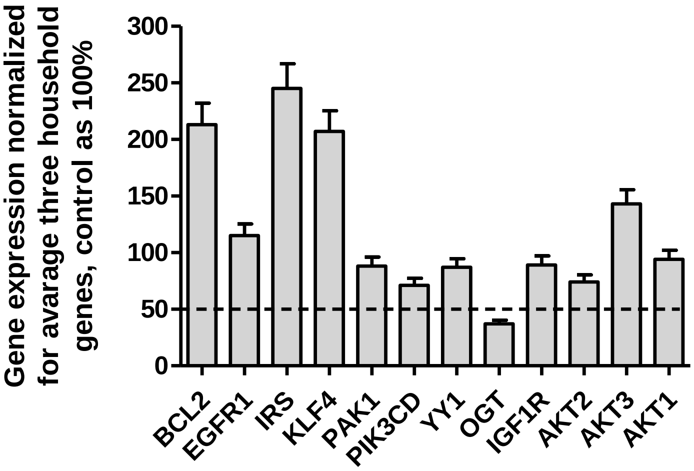

Figure S10: Modulated genes in HUVEC upon miR-7 transfection.HUVECs were transfected in the same way described in material & methods for RNA-seq analysis. 72 hrs post tranfection RNA of the cells was isolated using Trizol according to manufacturer's protocol. RT-PCR of miR-7 target genes according to literature indicated in the manuscript. Gene expression was normalized for average three household genes (HPRT, GAPDH, BGUS).

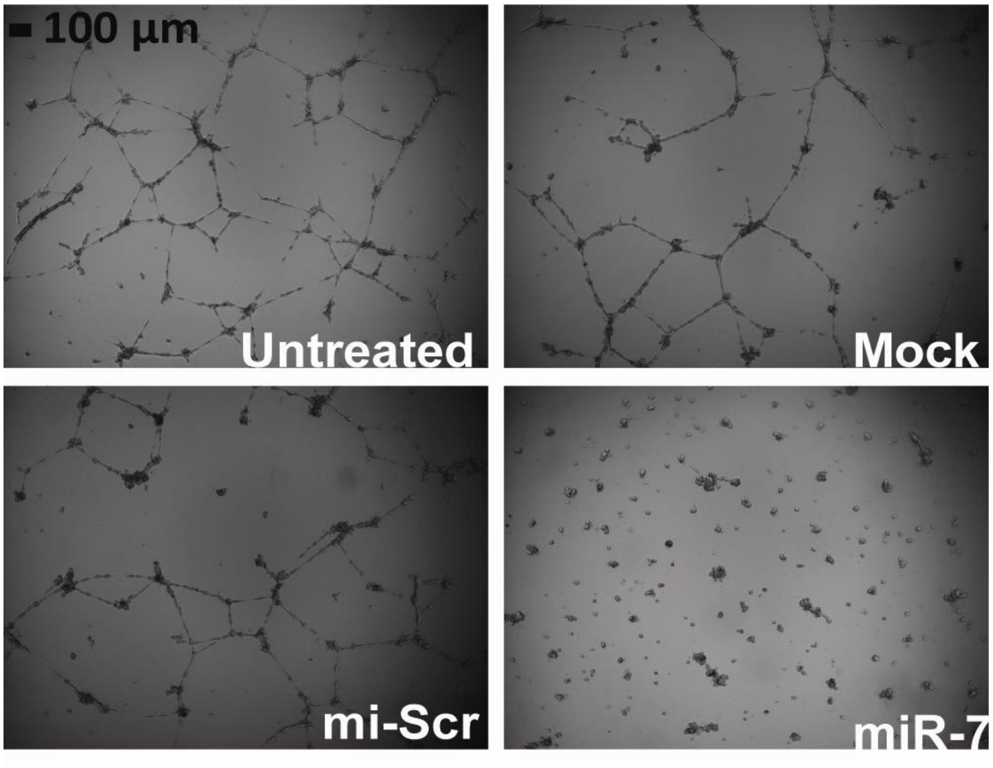

Figure S11: Magnification of Fig.1c two-dimensional tube formation of HUVEC after treatment with miR-7.

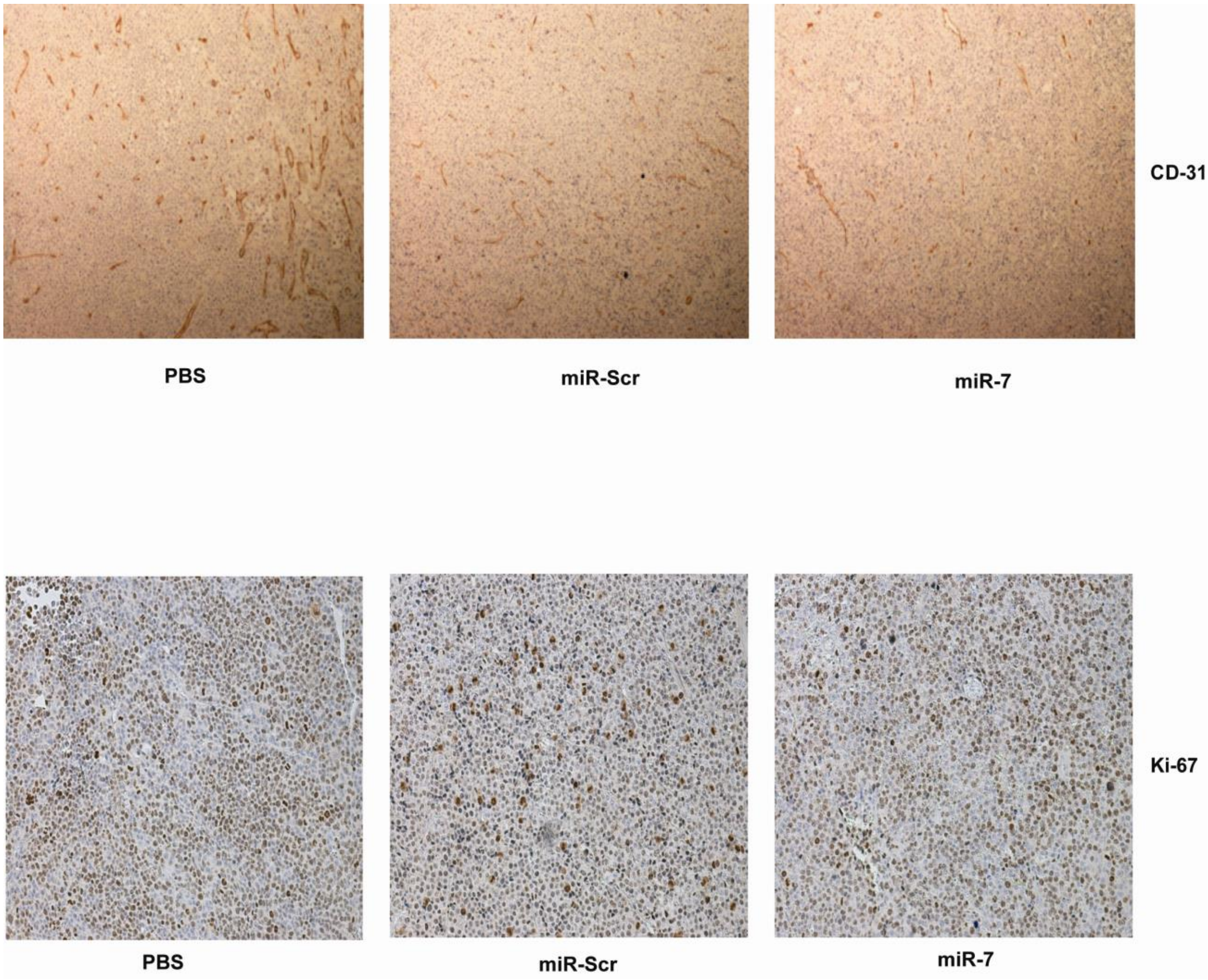

Figure S12: Magnification of Fig. 4c and e stained N2A tumor tissues against CD-31 and Ki-67.

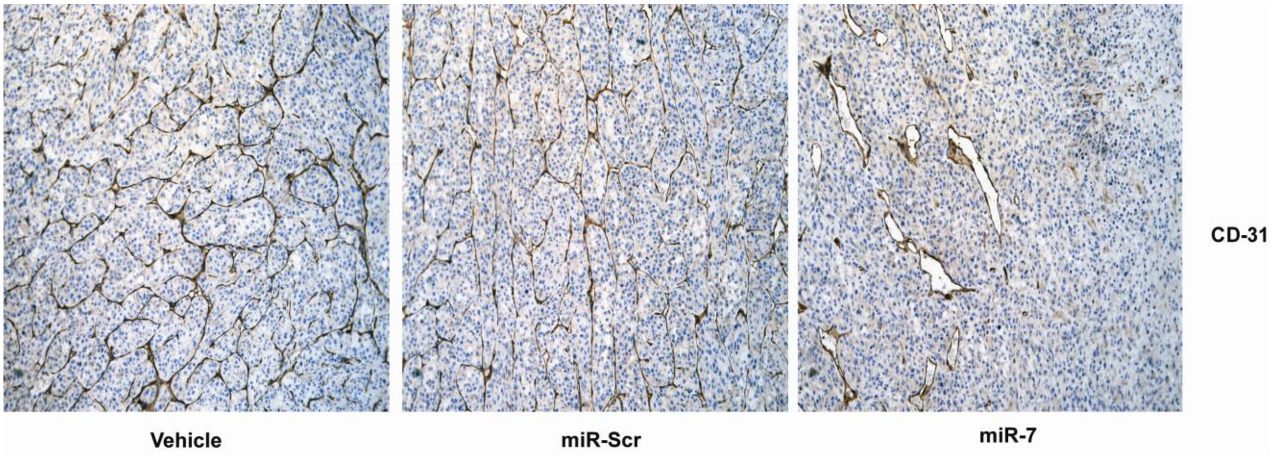

Figure S13: Magnification of Fig. 5c U-87 MG tumor tissues stained against CD-31.

Table S1: Percentage cell viability of 41 miRNA hits from the secondary screen in HUVEC and EC-RF24 using MTS read-out. The 41 miRNA hits were transduced in duplicate in HUVEC and EC-RF24 cells using two titers (MOI 100 and 200). Empty Vectors controls and non-responsive miRNAs from the secondary screen were used as negative controls. Mirrored plates were used to exclude plate effects. The average cell viability was calculated as percentage cell viability compared to Empty Vector control (100%). The anti-proliferative activity of the miRNAs was stronger in HUVEC than in EC-RF24 and therefore a cut-off value of less than 65% cell viability in HUVEC was used to come to a final list of 6 EC anti-proliferative miRNAs (Table 1).

	% of viability (MOI 100)		% of viability (MOI 200)	
**Lentivirus**	**HUVEC**	**EC-RF24**	**HUVEC**	**EC-RF24**
**hsa-miR-142**	52	88	46	81
**hsa-miR-190b**	63	75	58	72
**hsa-miR-26b**	60	72	53	57
**hsa-miR-302b**	143	117	146	137
**hsa-miR-574**	61	93	60	98
**hsa-miR-7-3 (*)**	45	53	41	43
**hsa-miR-9-2**	61	85	56	65
**hsa-miR-519d**	89	101	97	128
**hsa-miR-598**	83	98	89	93
**hsa-mir-302a**	99	95	91	77
**hsa-mir-27a**	95	88	87	84
**hsa-mir-92a**	128	127	84	119
**hsa-miR-940**	71	87	73	86
**hsa-miR-26a-1**	72	99	68	100
**hsa-miR-668**	76	110	76	95
**hsa-miR-766**	79	93	82	121
**hsa-miR-708**	76	114	81	109
**hsa-miR-16-2**	79	99	83	108
**hsa-miR-524**	88	93	99	96
**hsa-mir-30d**	95	91	96	90
**hsa-miR-1295**	89	98	92	90
**hsa-mir-370**	98	93	92	99
**hsa-miR-373**	98	89	100	118

**hsa-miR-653**	98	95	103	114
**hsa-miR-95**	95	103	93	98
**hsa-miR-639**	94	86	103	115
**hsa-miR-325**	93	95	101	97
**hsa-miR-1179**	88	92	90	79
**Candidate_001 (**)**	106	83	114	86
**Candidate_002**	95	87	93	110
**Candidate_003**	96	86	103	103
**Candidate_004**	93	91	96	107
**Candidate_005**	65	72	63	67
**Candidate_006**	68	85	66	102
**Candidate_007**	71	86	72	80
**Candidate_008**	71	86	79	92
**Candidate_009**	71	88	67	92
**Candidate_010**	89	91	100	82
**Candidate_011**	91	85	91	97
**Candidate_012**	93	97	92	101
**Candidate_013**	104	95	106	98

* The hsa-miR-7-3 family members, hsa-miR-7-1 and hsa-miR-7-2 did not pass the selection criteria of the primary screen. For selection criteria see Supplementary Fig. S1.

** Candidates are predicted miRNAs based on their genomic sequences and algorithms as described by Berezikov et al. (Genome Res. 2006, 10, 1289-1298). Over time many of the candidates in the library were annotated by miRBASE as true miRNA. Because these miRNAs originate from this proprietary dataset they were labeled as ‘candidate’.

Table S2: Expression of mature miRNAs after lentiviral transduction of miRNA in HUVEC.

HUVEC were seeded in a 24-well plate ($4x10^4$cells/well). Viral transduction was performed at MOI 50 according to protocol described in the legend of Supplementary Fig.S1. RNA isolation and RT-PCR was performed as described in Material and Methods.

Lentivirus	Mature miRNA	RT-PCR endogenous ($2^{-\Delta Ct}$)	RT-PCR ectopic ($2^{-\Delta Ct}$)	Overexpression (Fold)
**hsa-mir-142**	hsa-miR-142-3p	$1.08*10^{-7}$	$1.86*10^{-4}$	$1.73*10^{3}$
**hsa-mir-7-3**	hsa-miR-7-5p	$6.48*10^{-6}$	$1.61*10^{-3}$	$2.48*10^{2}$
**hsa-mir-26b**	hsa-miR-26b	$2.80*10^{-4}$	$2.09*10^{-3}$	7.47
**hsa-mir-574**	hsa-miR-574-5p	$8.26*10^{-5}$	$3.98*10^{-3}$	$4.82*10^{1}$
**hsa-mir-9-2**	hsa-miR-9	$1.47*10^{-5}$	$1.54*10^{-2}$	$1.05*10^{3}$
	hsa-miR-9*	$9.56*10^{-7}$	$2.74*10^{-3}$	$2.86*10^{3}$
**hsa-mir-190b**	hsa-miR-190b	$6.14*10^{-9}$	$4.27*10^{-3}$	$6.95*10^{5}$

Table S3: miR-7 associated regulated functions in HUVEC according to IPA software.

The set of 2500 miR-7 regulated genes in EC were loaded into IPA software (see Material and Methods). Based on the algorithm within IPA the gene expression profile was translated into cellular functions. When sufficient genes associated with a certain function are up or down-regulated, a cellular function is statistically up or down-regulated as reflected by a Z-score. All functions with as Z-score ≥ +2 or ≤ -2 were included in this Table.

Category	Functions Annotation	p-Value	Predicted Activation State	Regulation z-score	Number of Molecules affected
Cell Death	cell death	9.47E-19	Increased	2.324	581
Cell Death	apoptosis	5.12E-15	Increased	2.746	444
Cell Death	cell death of tumor cell lines	9.48E-14	Increased	2.368	252
Cell Death	apoptosis of tumor cell lines	1.40E-09	Increased	2.272	204
Organismal Survival	organismal death	2.45E-08	Increased	2.255	201
Cellular Assembly and Organization	development of cellular protrusions	9.66E-04	Increased	2.150	72
Cellular Assembly and Organization	neuritogenesis	2.03E-03	Increased	2.141	69
Tissue Development	neuritogenesis	2.03E-03	Increased	2.141	69
Nervous System Development and Function	neuritogenesis	2.03E-03	Increased	2.141	69
Cell Death	cell death of colon cancer cell lines	1.97E-03	Increased	2.030	43
Cell Death	cell death of carcinoma cell lines	1.76E-06	Increased	2.369	39
Cellular Assembly and Organization	extension of cellular protrusions	1.15E-04	Increased	2.370	36
Cellular Function and Maintenance	extension of cellular protrusions	1.15E-04	Increased	2.370	36
Cell Morphology	extension of cellular protrusions	1.15E-04	Increased	2.370	36
Cell Death	cell death of lung cancer cell lines	4.30E-04	Increased	2.950	36
Cellular Assembly and Organization	extension of plasma membrane projections	2.05E-03	Increased	2.760	28
Cellular Function and Maintenance	extension of plasma membrane projections	2.05E-03	Increased	2.760	28
Cell Morphology	extension of plasma membrane projections	2.05E-03	Increased	2.760	28
Cellular Assembly and Organization	extension of neurites	2.05E-03	Increased	2.402	26
Cellular Function and	extension of neurites	2.05E-03	Increased	2.402	26

Maintenance					
Cell Morphology	extension of neurites	2.05E-03	Increased	2.402	26
Nervous System Development and Function	extension of neurites	2.05E-03	Increased	2.402	26
Cellular Growth and Proliferation	proliferation of cells	7.51E-13	Decreased	-3.182	446
Gene Expression	expression of RNA	9.83E-04	Decreased	-2.147	319
Gene Expression	transcription	1.41E-03	Decreased	-2.149	293
Gene Expression	transcription of RNA	1.80E-03	Decreased	-2.303	287
Inflammatory Response	immune response	1.39E-04	Decreased	-3.037	227
Infectious Disease	infection by virus	7.66E-05	Decreased	-2.383	226
Cell Death	cell survival	1.41E-10	Decreased	-2.319	217
Infectious Disease	infection by Retroviridae	3.84E-04	Decreased	-2.655	168
Infectious Disease	infection by lentivirus	1.02E-03	Decreased	-2.539	164
Infectious Disease	HIV infection	1.65E-03	Decreased	-2.725	162
Cellular Growth and Proliferation	proliferation of tumor cell lines	9.12E-07	Decreased	-4.177	156
Infectious Disease	infection of cells	3.32E-04	Decreased	-2.457	143
Organismal Development	development of vessel	8.36E-09	Decreased	-2.208	130
Organismal Development	development of blood vessel	9.86E-09	Decreased	-2.301	129
Cardiovascular System Development and Function	development of blood vessel	9.86E-09	Decreased	-2.301	129
Cellular Movement	cell movement of leukocytes	2.48E-05	Decreased	-2.188	123
Hematological System Development and Function	cell movement of leukocytes	2.48E-05	Decreased	-2.188	123
Immune Cell Trafficking	cell movement of leukocytes	2.48E-05	Decreased	-2.188	123
Cancer	metastasis	3.66E-10	Decreased	-2.390	121
Cellular Movement	invasion of cells	6.85E-07	Decreased	-2.823	118
Organismal Development	vasculogenesis	2.02E-08	Decreased	-2.577	114
Cardiovascular System Development and Function	vasculogenesis	2.02E-08	Decreased	-2.577	114
Cellular Movement	cell movement of tumor cell lines	1.11E-05	Decreased	-2.525	112
Tissue Development	formation of tissue	1.75E-03	Decreased	-3.246	103
Cellular Movement	homing	6.50E-05	Decreased	-3.525	92
Nucleic Acid Metabolism	metabolism of nucleic acid component or derivative	1.88E-03	Decreased	-2.262	90
Cellular Movement	homing of cells	1.80E-04	Decreased	-3.544	87
Cancer	transformation	2.20E-04	Decreased	-2.716	87

Cellular Movement	cell movement of phagocytes	1.98E-04	Decreased	-2.774	85
Hematological System Development and Function	cell movement of phagocytes	1.98E-04	Decreased	-2.774	85
Immune Cell Trafficking	cell movement of phagocytes	1.98E-04	Decreased	-2.774	85
Inflammatory Response	cell movement of phagocytes	1.98E-04	Decreased	-2.774	85
Cellular Movement	chemotaxis	2.42E-04	Decreased	-3.476	85
Cellular Movement	cell movement of myeloid cells	5.17E-04	Decreased	-2.726	82
Hematological System Development and Function	cell movement of myeloid cells	5.17E-04	Decreased	-2.726	82
Immune Cell Trafficking	cell movement of myeloid cells	5.17E-04	Decreased	-2.726	82
Cancer	cell transformation	7.94E-04	Decreased	-2.620	82
Cellular Movement	chemotaxis of cells	5.36E-04	Decreased	-3.496	80
Cellular Movement	invasion of tumor cell lines	2.14E-04	Decreased	-2.268	79
DNA Replication. Recombination. and Repair	synthesis of DNA	7.79E-05	Decreased	-2.197	76
Small Molecule Biochemistry	metabolism of nucleotide	1.29E-03	Decreased	-2.271	76
Nucleic Acid Metabolism	metabolism of nucleotide	1.29E-03	Decreased	-2.271	76
Cellular Movement	migration of phagocytes	1.58E-04	Decreased	-2.145	49
Hematological System Development and Function	migration of phagocytes	1.58E-04	Decreased	-2.145	49
Immune Cell Trafficking	migration of phagocytes	1.58E-04	Decreased	-2.145	49
Inflammatory Response	migration of phagocytes	1.58E-04	Decreased	-2.145	49
Cellular Movement	cell movement of neutrophils	1.13E-03	Decreased	-2.194	48
Hematological System Development and Function	cell movement of neutrophils	1.13E-03	Decreased	-2.194	48
Immune Cell Trafficking	cell movement of neutrophils	1.13E-03	Decreased	-2.194	48
Inflammatory Response	cell movement of neutrophils	1.13E-03	Decreased	-2.194	48
DNA Replication. Recombination. and Repair	repair of DNA	1.52E-03	Decreased	-2.916	42
Cellular Growth and Proliferation	proliferation of smooth muscle cells	5.36E-04	Decreased	-2.038	41
Skeletal and Muscular System Development and Function	proliferation of smooth muscle cells	5.36E-04	Decreased	-2.038	41
Cellular Movement	migration of myeloid cells	3.33E-04	Decreased	-2.076	31
Hematological System Development and Function	migration of myeloid cells	3.33E-04	Decreased	-2.076	31
Immune Cell Trafficking	migration of myeloid cells	3.33E-04	Decreased	-2.076	31
Cell Cycle	S phase of tumor cell lines	7.06E-05	Decreased	-2.105	25

Cellular Assembly and Organization	orientation of chromosomes	9.45E-11	Decreased	-2.431	16
DNA Replication. Recombination. and Repair	orientation of chromosomes	9.45E-11	Decreased	-2.431	16
Cell Cycle	interphase of cervical cancer cell lines	1.19E-04	Decreased	-2.214	16
Cellular Assembly and Organization	alignment of chromosomes	5.78E-10	Decreased	-2.431	15
DNA Replication. Recombination. and Repair	alignment of chromosomes	5.78E-10	Decreased	-2.431	15
Cell Cycle	cycling of centrosome	1.56E-04	Decreased	-2.065	15
Cell Death	survival of fibroblasts	1.82E-03	Decreased	-2.894	15
Connective Tissue Development and Function	survival of fibroblasts	1.82E-03	Decreased	-2.894	15
Cellular Assembly and Organization	association of chromosome components	2.75E-06	Decreased	-2.361	11
Cancer	growth of carcinoma	1.72E-03	Decreased	-2.045	11
Cellular Assembly and Organization	chromosomal congression of chromosomes	2.39E-07	Decreased	-2.081	8
DNA Replication. Recombination. and Repair	chromosomal congression of chromosomes	2.39E-07	Decreased	-2.081	8
Cellular Assembly and Organization	association of chromatin	2.23E-05	Decreased	-2.436	8
Cell Death	cell viability of prostate cancer cell lines	2.14E-03	Decreased	-2.370	8
Lipid Metabolism	accumulation of glucosylceramide	7.84E-04	Decreased	-2.251	4
Small Molecule Biochemistry	accumulation of glucosylceramide	7.84E-04	Decreased	-2.251	4
Molecular Transport	accumulation of glucosylceramide	7.84E-04	Decreased	-2.251	4

Table S4: Concordance of downregulated angiogenesis associated genes with predicted miR-7 target genes.

Overlay of strongly down-regulated angiogenesis-associated genes (log2 ratio (miR-7 vs miR-Scr), *p*-value <0.05) with predicted miR-7 targets. OGT was the most strongly downregulated angiogenesis associated gene.

RNA-Seq		Gene name listed in case gene associated with function in Ingenuity	
**Gene in RNA-seq**	**Log2 ratio (miR-7 vs. miR-Scr)**	**Angiogenesis**	**Blood vessel formation**
**OGT**	-1.87	OGT	OGT
**KCNJ2**	-1.83		KCNJ2
**COL1A2**	-1.69		COL1A2
**CLIC4**	-1.69	CLIC4	CLIC4
**RB1**	-1.57		RB1
**CAV1**	-1.53	CAV1	CAV1
**RGS5**	-1.31	RGS5	
**CBL**	-1.30	CBL	CBL
**TFPI**	-1.29	TFPI	TFPI
**RAF1**	-1.13	RAF1	RAF1
**LEMD3**	-1.13		LEMD3
**ROCK2**	-0.96	ROCK2	ROCK2
**GATA6**	-0.91		GATA6
**BMPR2**	-0.87		BMPR2
**MIB1**	-0.87		MIB1
**GJC1**	-0.79		GJC1
**PSEN1**	-0.61		PSEN1

Table S5: Primers sequence

Primers	Sequence
SL_hsa-miR-7	5’-GTCGTATCCAGTGCAGGGTCCGAGGTATTCGCACTGGATAC GAACAACA-3'
forward_hsa-miR-7	5’-GCCCGCTTGGAAGACTAGTGATTTTG-3'
SL_hsa-miR-26b	5’-GTCGTATCCAGTGCAGGGTCCGAGGTATTCGCACTGGATAC GACACCTAT-3'
forward_hsa-miR-26b	5’-TGCCAGTTCAAGTAATTCAGGAT-3'
SL_hsa-miR-142-3p	5’-GTCGTATCCAGTGCAGGGTCCGAGGTATTCGCACTGGATAC GACTCCATA-3'
forward_hsa-miR-142-3p	5’-TGCCAGTGTAGTGTTTCCTACTTTA-3'
SL_hsa-miR-574-5p	5’-GTCGTATCCAGTGCAGGGTCCGAGGTATTCGCACTGGATAC GACACACAC-3'
forward_hsa-miR-574-5p	5’-TGCCAGTGAGTGTGTGTGTGTGAGT-3'
SL_hsa-miR-9	5’-GTCGTATCCAGTGCAGGGTCCGAGGTATTCGCACTGGATAC GACTCATAC-3’
forward_hsa-miR-9	5’-TGCCAGTCTTTGGTTATCTAGCTGT-3’
SL_hsa-miR-9*	5’-GTCGTATCCAGTGCAGGGTCCGAGGTATTCGCACTGGATAC GACACTTTC-3'
forward_hsa-miR-9*	5’-TGCCAGATAAAGCTAGATAACCGA-3'
SL_hsa-miR-190b	5'-GTCGTATCCAGTGCAGGGTCCGAGGTATTCGCACTGGATAC GAAACCCA-3'
Forward-miR-190b	5'-GCCCGCTTGATATGTTTGATATTG-3'
RT-PCR Reverse	5’-GTGCAGGGTCCGAGGT-3'
U6 stem loop primer	5’-GTCATCCTTGCGCAGG-3'
U6 forward primer	5’-CGCTTCGGCAGCACATATAC-3'
U6 reverse primer	5’-AGGGGCCATGCTAATCTTCT-3'
OGT forward primer	5’-ATCCTGATTTGTACTGTGTTCGC-3’
OGT reverse primer	5’-CAGGGCTTTGAGCAGGTTC-3’
HPRT1 forward primer	5’-CCTGGCGTCGTGATTAGTGAT-3’
HPRT1 reverse primer	5’-AGACGTTCAGTCCTGTCCATAA-3’
GAPDH forward primer	5’-AAGGTGAAGGTCGGAGTCAAC-3’
GAPDH reverse primer	5’- GGGGTCATTGATGGCAACAATA-3’
GUSB forward primer	5’- GAAAATATGTGGTTGGAGAGCTCATT-3’
GUSB reverse primer	5’- CCGAGTGAAGATCCCCTTTTTA-3’
BCL2 forward primer	5’-GCCTTCTTTGAGTTCGGTGG-3’
BCL2 reverse primer	3’-ATCTCCCGGTTGACGCTCT-5’
IRS forward primer	5’-ACACCTACGCCAGCATTGAC-3’
IRS reverse primer	3’-CTTCGGGCTGAAACAGTGCT-5’
KLF4 forward primer	5’-CCACACTTGTGATTACGCGG-3’
KLF4 reverse primer	3’-TACGGTAGTGCCTGGTCAGT-5’
PAK1 forward primer	5’-TTCCGGGACTTTCTGAACCG-3’
PAK1 reverse primer	3’-AGAGGGGCTTGGCAATCTTC-5’
PIK3CD Forward primer	5’-AAGGAGGAGAATCAGAGCGTT-3’
PIK3CD forward primer	3’-GAAGAGCGGCTCATACTGGG-5’
AKT1 forward primer	5’-CAGGATGTGGACCAACGTGA-3’

AKT1 reverse primer	3’-AAGGTGCGTTCGATGACAGT-5’
AKT2 forward primer	5’-CAAGCGTGGTGAATACATCAAGA-3’
AKT2 reverse primer	3’-GCCTCTCCTTGTACCCAATGAA-5’
AKT3 forward primer	5’-TGTGGATTTACCTTATCCCCTCA-3’
AKT3 reverse primer	3’-GTTTGGCTTTGGTCGTTCTGT-5’
YY1 forward primer	5’-TCAGATCCCAAACAACTGGCA-3’
YY1 reverse primer	3’-GGCCGAGTTATCCCTGAACA-5’
EGFR forward primer	5’-TTGCCGCAAAGTGTGTAACG-3’
EGFR reverse primer	3’-TCACCCCTAAATGCCACCG-5’

Table S6: 3’UTR sequence

OGT 3’UTR sequence with miR-7 binding site (blue, predicted by microRNA.org, underlined is seed)

TGGGGGAAAGGGAACTAGATAACATACTTCTTACTTGTCTGTACAGTACCTTGTTGC
AGATGGGTGATATATAATGGTAATAGAATAGCACAGCCAGACTTGCTTCCTGCATGG
TAGGGAGAGACACAAAAGATGGGAAACTGCTTTTCCACAAGGAATCTCCGTAGAAT
TTTGCGGCGACCAGATGGTGCATAGGTCTGGAAGGTCTGATCTCCCTTGGTCTTCCA
TGGGATGGTTAGTGTGGAGGGGAGATATAGATTGTCCGGCCGCTTTGTGATTCCATG
GATTGATTCAGTCTTCTGGATTTTTTTTTCTTTATATTTTGGGTACTGGAGCTTTTAAA
AATGTTTGGTTTCAGGTATTTTTATTCATGTGAAGTGTATATGATTCTCTTGAGATAA
GGTTTTAAGCTAAAATGTTACTCCCTGTT

OGT 3’UTR sequence with mutation with miR-7 binding site (blue, predicted by microRNA.org, underlined is seed, mutated nucleotides in bold)

TGGGGGAAAGGGAACTAGATAACATACTTCTTACTTGTCTGTACAGTACCTTGTTGC
AGATGGGTGATATATAATGGTAATAGAATAGCACAGCCAGACTTGCTTCCTGCATGG
TAGGGAGAGACACAAAAGATGGGAAACTGCTTTTCCACAAGGAATCTCCGTAGAAT
TTTGCGGCGACCAGATGGTGCATAGGTCTGGAAGGTCTGATCTCCCTTGGTCT**AG**CA
TGGGATGGTTAGTGTGGAGGGGAGATATAGATTGTCCGGCCGCTTTGTGATTCCATG
GATTGATTCAGTCTTCTGGATTTTTTTTTCTTTATATTTTGGGTACTGGAGCTTTTAAA
AATGTTTGGTTTCAGGTATTTTTATTCATGTGAAGTGTATATGATTCTCTTGAGATAA
GGTTTTAAGCTAAAATGTTACTCCCTGTT

Table S7: Differential expression of miR-7 literature target genes in EC.

The log2 ratio was calculated as described in Material and Methods and reflects the differential expression of genes in HUVEC treated with miR-7 and gene expression in HUVEC treated with miR-Scr.

Target Genes	References	Tumor models	RNA-seq Log2 of gene expression ratio (miR-7 vs. miR-Scr), $p<0.05$
**Akt**	(Kefas. Godlewski et al. 2008) (Fang. Xue et al. 2012)	Glioblastoma Hepatocellular Carcinoma	-
**BCL2**	(Xiong. Zheng et al. 2011)	Lung cancer	-
**EGFR-1**	(Kefas. Godlewski et al. 2008) (Webster. Giles et al. 2009) (Kalinowski. Giles et al. 2012)	Glioblastoma Lung cancer Breast cancer Head & Neck Cancer	-
**IGF1R**	(Zhao. Dou et al. 2013)	Gastric cancer	-
**IRS**	(Kefas. Godlewski et al. 2008) (Giles. Brown et al. 2013)	Glioblastoma Melanoma	-
**KLF4**	(Okuda. Xing et al. 2013)	Brain cancer	-
**Pak1**	(Reddy. Ohshiro et al. 2008)	Breast Cancer	-
**PIK3CD**	(Fang. Xue et al. 2012)	Hepatocellular Carcinoma	-
**YY1**	(Zhang. Li et al. 2012)	Colorectal cancer	-

Fang. Y.. J. L. Xue. et al. (2012). "MicroRNA-7 inhibits tumor growth and metastasis by targeting the phosphoinositide 3-kinase/Akt pathway in hepatocellular carcinoma." Hepatology **55**(6): 1852-1862.

Giles. K. M.. R. A. Brown. et al. (2013). "miRNA-7-5p inhibits melanoma cell migration and invasion." Biochem Biophys Res Commun **430**(2): 706-710.

Kalinowski. F. C.. K. M. Giles. et al. (2012). "Regulation of epidermal growth factor receptor signaling and erlotinib sensitivity in head and neck cancer cells by miR-7." PLoS One **7**(10): e47067.

Kefas. B.. J. Godlewski. et al. (2008). "microRNA-7 inhibits the epidermal growth factor receptor and the Akt pathway and is down-regulated in glioblastoma." Cancer Res **68**(10): 3566-3572.

Okuda. H.. F. Xing. et al. (2013). "miR-7 suppresses brain metastasis of breast cancer stem-like cells by modulating KLF4." Cancer Res **73**(4): 1434-1444.

Reddy. S. D.. K. Ohshiro. et al. (2008). "MicroRNA-7. a homeobox D10 target. inhibits p21-activated kinase 1 and regulates its functions." Cancer Res **68**(20): 8195-8200.

Xiong. S.. Y. Zheng. et al. (2011). "MicroRNA-7 inhibits the growth of human non-small cell lung cancer A549 cells through targeting BCL-2." Int J Biol Sci **7**(6): 805-814.

Zhang. N.. X. Li. et al. (2012). "microRNA-7 is a novel inhibitor of YY1 contributing to colorectal tumorigenesis." Oncogene.

Zhao. X.. W. Dou. et al. (2013). "MicroRNA-7 functions as an anti-metastatic microRNA in gastric cancer by targeting insulin-like growth factor-1 receptor." Oncogene **32**(11): 1363-1372.
